# Phenotypic heterogeneity and tumor immune microenvironment directed therapeutic strategies in pancreatic ductal adenocarcinoma

**DOI:** 10.3389/fimmu.2025.1573522

**Published:** 2025-03-31

**Authors:** Remya P. G. Ramesh, Hadida Yasmin, Pretty Ponnachan, Basel Al-Ramadi, Uday Kishore, Ann Mary Joseph

**Affiliations:** ^1^ Department of Veterinary Medicine, UAE University, Al Ain, United Arab Emirates; ^2^ Immunology and Cell Biology Laboratory, Department of Zoology, Cooch Behar Panchanan Barma University, Cooch Behar, West Bengal, India; ^3^ Department of Medical Microbiology and Immunology, College of Medicine and Health Sciences, United Arab Emirates University, Al Ain, United Arab Emirates; ^4^ Zayed Center for Health Sciences, United Arab Emirates University, Al Ain, United Arab Emirates; ^5^ ASPIRE Precision Medicine Research Institute Abu Dhabi, United Arab Emirates University, Al Ain, United Arab Emirates

**Keywords:** pancreatic cancer, tumor microenvironment, tumor heterogeneity, immunotherapy, desmoplastic stroma, immunosuppression

## Abstract

Pancreatic cancer is an aggressive tumor with high metastatic potential which leads to decreased survival rate and resistance to chemotherapy and immunotherapy. Nearly 90% of pancreatic cancer comprises pancreatic ductal adenocarcinoma (PDAC). About 80% of diagnoses takes place at the advanced metastatic stage when it is unresectable, which renders chemotherapy regimens ineffective. There is also a dearth of specific biomarkers for early-stage detection. Advances in next generation sequencing and single cell profiling have identified molecular alterations and signatures that play a role in PDAC progression and subtype plasticity. Most chemotherapy regimens have shown only modest survival benefits, and therefore, translational approaches for immunotherapies and combination therapies are urgently required. In this review, we have examined the immunosuppressive and dense stromal network of tumor immune microenvironment with various metabolic and transcriptional changes that underlie the pro-tumorigenic properties in PDAC in terms of phenotypic heterogeneity, plasticity and subtype co-existence. Moreover, the stromal heterogeneity as well as genetic and epigenetic changes that impact PDAC development is discussed. We also review the PDAC interaction with sequestered cellular and humoral components present in the tumor immune microenvironment that modify the outcome of chemotherapy and radiation therapy. Finally, we discuss different therapeutic interventions targeting the tumor immune microenvironment aimed at better prognosis and improved survival in PDAC.

## 1 Introduction

**Figure 1:**
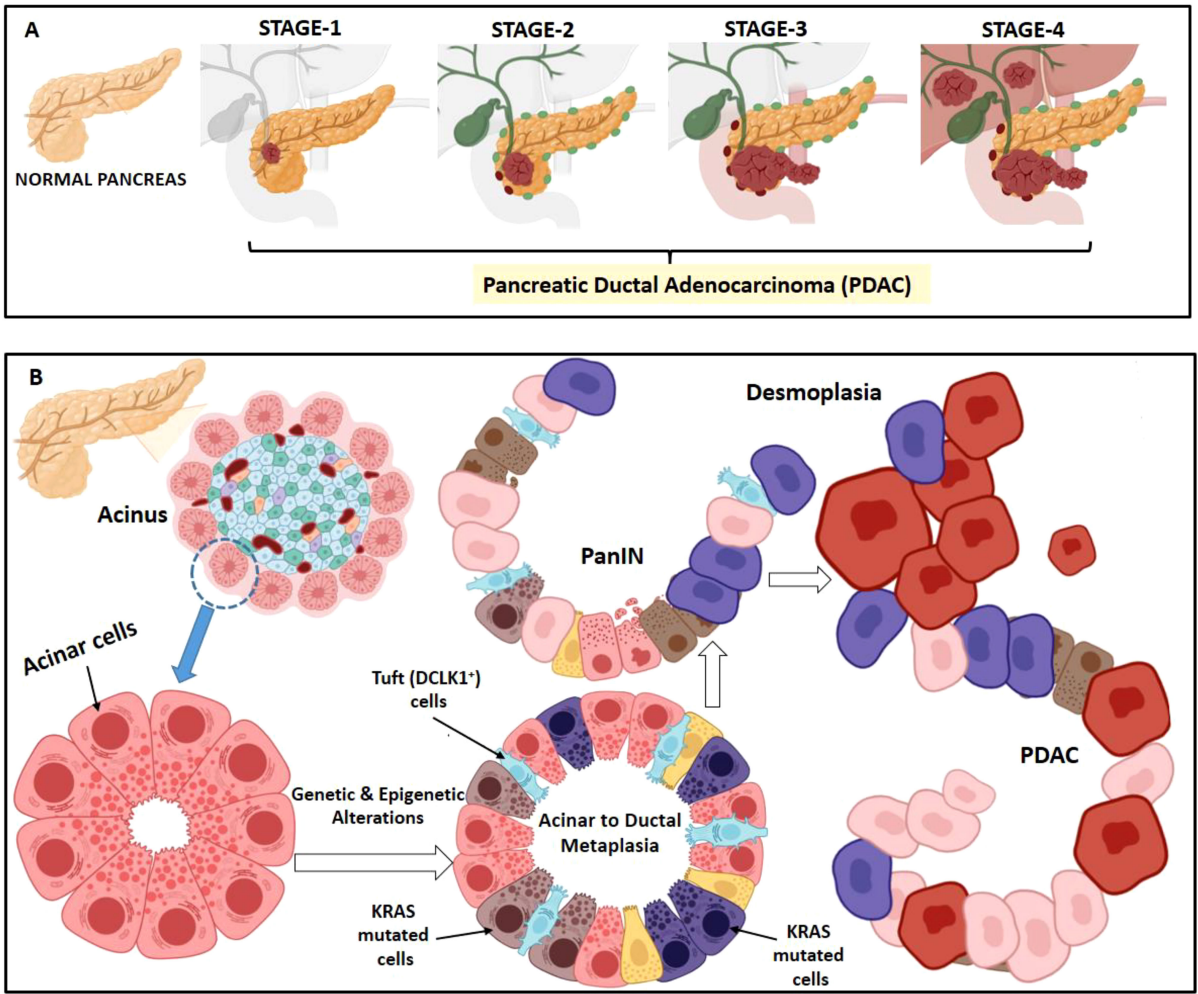
Stages in PDAC development. **(A)** PDAC can be pathologically categorized on the basis of TNM staging system i.e. size and extent of the tumor, spread to lymph nodes and metastasis. Stage 1 is when the tumor is restricted to the pancreas; stage 2 when the tumor has spread to 2 or 3 nearby lymph nodes; stage 3, when the tumor has spread to 4 or more nearby lymph node and may have also reached nearby blood vessel; stage 4, when the cancer has metastasized in other organs (liver, lungs etc.). **(B)** Acinar to ductal metaplasia is a normal regeneration process happening in pancreas during inflammation. However, due to KRAS hyperactivation, acinar cells fail to redifferentiate and progresses to duct-like cells forming pancreatic intraepithelial neoplasia (PanIN). PanINs are microscopic papillary or flat non-invasive epithelial neoplasms arising in pancreatic ducts characterized by mucin-containing cuboidal to columnar cells. With accumulation of subsequent additional mutations, the cancer progresses to a demoplasia condition causing PDAC.

Pancreatic Ductal Adenocarcinoma (PDAC) is the predominant form of aggressive pancreatic cancer, characterized by high mortality rates, originating from the ductal cells that form the pancreatic ducts (1). Microscopically margin-free surgical removal of the pancreas is the only viable and potentially curative method. The majority of individuals exhibit this condition at a non-resectable stage, reinforcing the necessity for early diagnosis and identification. Unfortunately, aggressive disease progression and early metastasis contribute to late diagnosis, with less than 20% of patients presenting with a resectable tumor at diagnosis (2). In addition, phenotypic heterogeneity in PDAC refers to the variation in cell types and characteristics within a tumor. This heterogeneity is a significant factor in the disease progression, chemotherapy resistance, and overall prognosis. The metastatic potential is also extremely high and tumors spread mainly through lymphatic and blood vessels. Most of the patients already have metastasis in the liver and lymph nodes at the time of diagnosis (3, 4) (**Figure 1A**). Recent reviews on PDAC heterogeneity, tumor immune microenvironment (TiME), and emerging therapies shed light into the current research (5–7). We intend to provide an understanding about PDAC and the molecular and immune landscape affecting various therapeutic interventions. Then we discuss the difficulties associated with phenotypic heterogeneity affecting chemotherapy, adjuvant therapies and immunotherapy.

## 2 Stages in PDAC development

In pancreas, acinar to ductal metaplasia is a usually reversible phenomenon of healing upon injury or inflammation. However, this becomes irreversible due to accumulation of KRAS mutation. This KRAS hyperactivation in acinar cells makes them incapable to redifferentiate and puts a halt at the duct-like cell stage leading to pancreatic intraepithelial neoplasia (PanIN) (8, 9) (**Figure 1B**). PDAC exhibits genetic heterogeneity and presents with several clinical characteristics associated with epithelial neoplasms. Patient stratification is essential based on histology and molecular characteristics for effective therapy. The three well established precursor lesions of pancreatic cancer includes PanIN, intraductal papillary-mucinous neoplasm (IPMN) and mucinous cystic neoplasm (MCN) (10, 11) Atypical flat lesion is yet another precursor (12). PanINs are microscopic papillary or flat non-invasive epithelial neoplasms arising in pancreatic ducts characterized by mucin-containing cuboidal to columnar cells. IPMNs are tumors of the duct epithelium characterized by mucin-producing epithelial neoplasms, usually with papillary architecture. They arise from the main pancreatic duct or branch ducts. Activating KRAS mutations are observed in 50% of IPMNs; its prevalence increases as does the degree of dysplasia (13).

Oncogenic KRAS mutations are the most common genomic alterations identified in PDAC followed by tumor protein p53 (TP53), cyclin-dependent kinase inhibitor 2A (CDKN2A), and suppressors of mothers against decapentaplegic homolog 4 (SMAD4) (14, 15). Progression from low grade PanIN-1 to PanIN-2 and high grade PanIN-3 lesions are with 45% incidence of *KRAS* mutations in PanIN-1 stage along with telomere shortening in 90% of cases (16, 17). Overexpression of another PDAC associated gene, p21(WAF1/CIP1) occurs early in the development of PanIN (18). Inactivation of *CDKN2A* and *TP53* is found in IPMNs with high grade dysplasia, loss of SMAD4 is observed in a small subset. Prevalence of *KRAS* mutations and aberrant nuclear TP53 accumulation is noted with increasing degree of dysplasia in MCN (19). With gradual acquisition of gene mutations, the cancer progresses with increase in desmoplasia, a condition of excessive formation of fibrous connective tissue causing PDAC.

### 2.1 PDAC subtypes

Transcriptional profiling using microdissected PDAC samples has led to identification of major molecular subtypes, namely classical, quasi-mesenchymal and exocrine-like (20). The classical subtype (CLA) showed high expression of epithelial and adhesion-associated genes, such as the transcription factor *GATA6*; this is associated with a favorable prognosis in terms of survival following PDAC resection. Quasi-mesenchymal subtype exhibits high expression of mesenchymal-associated genes, relatively less dependent on KRAS than CLA and is associated with poor prognosis. Finally, the exocrine-like subtype revealed high expression of digestive exocrine enzyme genes (21). Moffit’s classification identified two tumor specific subtypes, Basal like (BL), CLA subtype and two stromal subtypes (22). BL shows poor prognosis and therapy resistance to gemcitabine-based chemotherapy and FOLFIRINOX; CLA subtype is responsive to erlotinib, an EGFR antagonist (23–25). Puleo et al. classified PDAC into five subtypes: Pure-basal-like, Stroma-activated, Desmoplastic, Pure-classical, and Immune-classical (26). This classification was based on the influence of TME and the tumor cells. These PDAC subtypes have characteristics of both cancerous and immunological cells that may be sensitive to drug therapy. Espinet et al. explored the molecular mechanisms driving subtype heterogeneity in PDAC and its influence on therapy response. It highlights the role of immune cells, transcriptional networks, and epigenetic factors in shaping PDAC subtypes, particularly in BL subtype with therapy resistance (27). Growing evidence supports the coexistence of BL and CLA subtypes (28). A significant proportion of tumors comprises cells that co-express CLA and BL markers, thereby creating a continuum between these two phenotypes, due to cytokine gradients secreted by tumor and stromal cells in a paracrine manner within various spatially distinct microenvironments (29–31).

## 3 Genomic and epigenomic aspects

### 3.1 Activating and inactivating mutations

The Cancer Genome Atlas (TCGA) pancreatic cancer study reveals a low burden of tumor mutation, typically < 50 mutations; only a few cases showing > 80 mutations. However, recurrent mutations occurring in certain cancer-associated genes are the main cause of the disease pathogenesis (32). The most common activating mutation that occurs in approximately 65.4% of pancreatic cancer cases is KRAS mutation (32, 33). The KRAS mutation that initiates PDAC takes place over a period of 12 years (34).

Molecular pathogenesis in PDAC is marked by the alterations in the canonical genes. Genetic analyses indicate that PDAC tumors harbor multiple high-potency oncogenic and tumor suppressive lesions. Whole genome sequencing revealed that genetic aberrations lead to the development of PDAC. Approximately 90% of PDAC patients have KRAS mutations (35), resulting in the formation of pancreatic lesions called PanIN. 50-74% have inactivating mutations in TP53, 46-60% in CDKN2A mutations, and ~31–38% have mutations in SMAD4, which can occur at the late stages (36, 37). Activation of oncogenic KRAS (38) or inactivation of tumor suppressor TP53 (39), SMAD4 (40) or CDKN2A (41), Krüppel-like Factor 5 (KLF5), is increased in PDAC which promotes proliferation and also acinar-to-ductal metaplasia, PanIN, and leads to tumor growth in mice (42). Genetic analysis has provided insights into the altered signaling pathways (43).

The KRAS gene encodes a small GTPase, acting as a molecular switch cycling between an active GTP-bound and inactive GDP-bound state for various cellular processes, proliferation and survival. KRAS protein, once bound to GTP, is capable of interacting with downstream proteins and activates the effector signaling pathways that drives cancer growth. Thus, the mutation in KRAS gene leads to impairment of GTPase activity hampering other signaling pathways, including inactivation of tumor suppressor pathways (44–46). KrasG12D mutation alone leads to formation of PanIN (47, 48). In addition to KRAS, the mutational inactivation of TP53 (49), *SMAD4* (50) and *CDKN2A* (47, 51) tumor suppressor genes leads to progression of PanIN formation to rapid and high-frequency metastatic PDAC.

CDKN2A gene codes for cell cycle inhibitors, p16 signalling and SMAD4, which act as transducers of the transforming growth factor beta (TGF-β) signalling pathway, and are two commonly mutated tumor suppressors found in about 20% of PDAC cases (52). *CDKN2A* encodes p16^INK4A^ and p19^ARF^ through a common locus on chromosome 9p (53). p16^INK4A^ is a cell cycle inhibitor acting at the G1-S checkpoint through *cyclin*-*dependent kinases* (*CDKs*) (54), whereas p19^ARF^ binds to mouse double minute 2 homolog (*MDM2*), an *E3 ubiquitin*-protein *ligase* inhibiting p53 degradation and thus, causes cell cycle arrest independent of CDKs (55). Large homozygous deletions, missense mutations, promoter methylation of CDKN2A along with promoter silencing, inactivates both these protein (p16^INK4A^ and p19^ARF^), resulting in almost universal loss of CDKN2A leading to PDAC (33).

Approximately 50-74% of pancreatic cancers have inactivating mutations in TP53, the most frequently detected genetic alteration (56). About 66% of *TP53* mutations are missense affecting its DNA binding domain (43, 56); nonsense mutations, frameshifts and homozygous deletions are considered vital mechanisms of *TP53* inactivation in PC (43). In late-stage pancreatic cancers, almost 50% of all mutations in TP53 cause loss of protein expression leading to null alleles (56). Integrated mutation profiling of actionable cancer targets (MSK-IMPACT study) and the GENIE project suggests mutations of *TP53* in about 70% PDAC subsets (52). p53 mutations are observed in some PanIN lesions, but not in IPMN, developing at the later stage where two-thirds of the mutations are missense mutations (57).

*SMAD4* or DPC4 (deleted in pancreatic cancer locus 4) gene that is inactivated in about 60% of PDAC cases in late stages, is an intracellular transcriptional mediator of the TGF-β signaling pathway (58, 59). TGF-β is one of the most commonly mutated signal transduction pathway in PDAC (60, 61). However, inactivating mutation in TGFβ receptor 1 (*TGFBR1), TGFBR2*, activin A receptor type 1B (*ACVR1B*) or *SMAD3* has been identified in about 10% cases, possibly via inhibiting TGF-β signaling (14, 43). KLF11 is a transcription factor that increases TGF-β signaling by inhibiting Smad7, a negative regulator of the pathway. This facilitates growth inhibition and tumor suppression in normal epithelial cells. In pancreatic cancer cells harboring oncogenic Ras mutations, the Erk-MAPK pathway phosphorylates KLF11, thereby impairing its association with mSin3A corepressor and inhibiting Smad7 suppression. The inactivation of KLF11 results in diminished TGF-β signaling, which facilitates the growth of pancreatic cancer (62).

A study involving 142 pancreatic cancer patients, using next-generation sequencing platforms showed heterogeneity among 2,016 non-silent mutations and 1,628 copy-number variations (14). Sixteen mutated genes, along with KRAS, CDKN2A and TP53, were identified. They are Myeloid/lymphoid or mixed-lineage leukemia protein 3 (*MLL3)*, AT-rich interactive domain-containing protein 1A *(ARID1A)*, AT-rich interactive domain-containing protein 2 (*ARID2)*, Transforming growth factor-β receptor type II (*TGFBR2)*, and Splicing Factor 3b Subunit 1 (*SF3B1)*, Enhancer of polycomb homologue 1 (*EPC1)*, Dual specificity mitogen-activated protein kinase kinase 4 (MAP2KA), Ataxia telangiectasia mutated (ATM), Zinc finger imprinted 2 (ZIM2), Sodium leak channel non-selective protein (*NALCN)*, Melanoma-associated antigen 6 (MAGEA6), and Solute carrier family 16 member 4 (*SLC16A4*). Somatic aberrations in genes guiding axons in SLIT/ROBO signaling were also observed (14, 24). *ATM* (gene product mutated in human genetic disorder ataxia telangiectasia) is one of the most commonly mutated DDR genes which is sporadically mutated in familial pancreatic cancer (63). ATM encodes for PI3K-related serine/threonine protein kinase capable of repairing DNA double-strand breaks, have been identified with somatic mutations in 2 to 18%, and 1 to 34% of germline mutations in PDAC patients (64).

To avoid cross-contamination of tumor cells with non-neoplastic cells, prior to sequencing, each tissue sample was first immunolabeled for the proteins encoded by most commonly altered tumor-suppressor genes inactivated in PDAC (CDKN2A, TP53, and SMAD4) (65). The activating mutations of KRAS are almost ubiquitous and inactivation of TP53, SMAD4 and CDKN2A occur at the rates of >50%. Missense mutations were identified in TP53, ARID1A and in SMAD4 corresponding to the immunolabeling patterns which were uniformly present in all samples studied (65). Whole-genome sequencing and copy number variation (CNV) analysis of 100 PDAC samples suggested prevalence of gene disruption due to chromosomal rearrangements, which affected TP53, SMAD4, CDKN2A, ARID1A and ROBO2 (37). The study showed a total of 857,971 somatic point mutations with small insertions and deletions, where 5,424 genes exhibited a total of 7,888 non-silent mutations. Out of 11,868 somatic structural variants, 10114 were intra-chromosomal and 1754 inter-chromosomal translocations. In the case of intra-chromosomal locations, 5860 showed intrachromosomal rearrangements, 1629 inversions, 1393 deletions, 579f inversions, 346 amplified inversions, 128 duplications, and 179 tandem duplications (37). This study came up with 4 subtype classification of PDACs, on the basis of variations in chromosomal structure with potential clinical relevance; stable (subtype-1), locally rearranged (Subtype-2), scattered (subtype-3) and unstable (subtype-4). In case of subtype-1, the tumor genomes had ≤ 50 structural variation events and often showed aneuploidy. In subtype-2, there were rearranged genomes with regions of amplifications containing known oncogenes such as KRAS, SOX9 and GATA6, in addition to genes of therapeutic targets such as ERBB2, MET, CDK6, PIK3CA and PIK3R3, among 1–2% of patients. This subtype also showed complex genomic rearrangement events such as breakage–fusion–bridge or chromothripsis resulting in a ring chromosome. In 36% of samples (subtype-3), a moderate range of non-random chromosomal damage was observed. The unstable type (subtype-4) seen in 14% of samples showed high genomic instability with >200 structural variation events, suggesting sensitivity to DNA-damaging agents. Mutations in BRCA pathway genes accounted for approximately half of the patients with a high BRCA mutational signature and overlapping deleterious mutations in BRCA1, BRCA2 and PALB2 with unstable genomes pointing towards deficiencies in DNA damage repair (37).

### 3.2 Circulating DNA markers

Different types of circulating DNA are traced in liquid biopsies, such as total circulating cell free cfDNA, circulating tumor-specific cell-free DNA (ctDNA), mRNA, and microRNA (miRNA) containing tumor-derived markers. cfDNA is double-stranded fragmented DNA found at a low level under physiological concentrations, but its level increases under chronic and acute pathological conditions including cancers (66). The concentration of cfDNA in blood ranges between 0–5 and >1000 ng/ml in cancer patients while in the range of 0 and 100 ng/ml in healthy individuals (67).

cfDNA, which are greater than 10 kb, are mostly released in the blood or in body fluids from healthy cells of hematopoietic origin, or from cancer cells probably due to apoptosis and necrosis of tumor tissue during cancer treatment (68). However, shorter fragments of DNA (<100bp) mostly comprises of ctDNA, mitochondrial DNA (mtDNA), and bacterial DNA. ctDNA is single or double-stranded DNA harboring tumor specific somatic mutations, and thus, its use as a biological marker has drawn much attention and considered a promising prognostic factor for PDAC (69, 70). Its smaller size makes them difficult to detect and quantify. The half-life of ctDNA ranges between 16 and 150 mins, and thus, also provides lower sensitivity during early detection in liquid biopsies (67, 70–72). The presence of ctDNA in the blood has been linked to relapse and residual disease after PDAC surgery, possibly due to surgery- induced injury to the tumor tissue releasing the DNA (72–74).

In a study to investigate whether ctDNA could identify minimal residual disease (MRD) and predict relapse of PDAC after surgery, panel-captured sequencing was performed to detect somatic mutations in 27 patients and 65 plasma samples (75). ctDNA was detected in 18 of 27 preoperative plasma samples, resulting in a detectable rate of 66.67% and reaching 100% for stage IV PDAC. Nine patients were positive for post-operative ctDNA and had a markedly reduced disease-free survival (DFS) with ctDNA-negative ones. Compared to matched tumor tissue, the frequency of mutant genes was lower in ctDNA, suggesting poor shedding into peripheral blood in PDAC patients and low allelic frequency that is hard to detect considering the genomic heterogeneity and multiple mutations in PDAC. However, about 90% recurrence patients were postoperative ctDNA-positive, suggesting sensitivity of postoperative ctDNA analysis than CT scanning in MRD identification (75).

Study of serum samples from 61 metastatic PDAC patients suggested higher association of cfDNA concentration (median concentration being 33 ng/mL) and fragmentation (100–1100 bp) levels with poorer survival in metastatic PDAC (76).

5-methylcytosine (5mC) and 5-hydroxymethylcytosine (5hmC) markers from cfDNA have recently been considered as important markers for non-invasive diagnosis for PDAC. A recent study involving high throughput sequencing (cfMeDIP-seq) and cell-free 5hmC sequencing methods, global loss of 5hmC in the PDAC samples was observed by Integrative Genomics Viewer (77). 5hmC enriched regions were high in coding DNA sequence (CDS), 5′UTRs, exons, 3′UTRs, and promoters, but introns and intergenic regions were depleted in 5mC as well as 5hmC. Upon comparing the distribution, 5mC peaks were found two-fold higher than 5hmC peaks where only 16.7% overlapped with 5hmC peak sites. The overlapping peaks were in five types of histone modifications (H3K36me3, H3K27ac, H3K4me1, H3K4me3, and H3K27me3). Over 80% of 5mC peaks occurred at sites distinct to 5hmC, where 17,340 genes carried both the types of modifications. Combining both 5mC and 5hmC features and paired datasets from PDAC and healthy samples, around 51 features were found that could discriminate PDAC from healthy samples and disparity of the weighted diagnosis score (wd-score) was statistically significant. The wd-score with the 5hmC model could also distinguish stage I patients from stage II–IV PDAC patients (77).

A significant global decrease in 5hmC signal was seen in PDAC compared to non-cancer cohort, which increased over 3’UTR, transcription termination sites (TTS) and intron regions and decreased over promoters (78). Few studies also suggested overlap in gene centric functional regions such as in promoters, untranslated regions (3′UTRs and 5′ UTRs), TTS, exons and short interspersed repetitive elements that increased in comparison to genomic background. In almost all PDAC stages, 5hmC decrease over H3K4me3-marked active TSS sites was noted. Among top 50 hyperhydroxymethylated and hypohydroxymethylated genes, PDAC tissue-derived hyper-hydroxymethylated genes were capable of discriminating between non-cancer and PDAC cfDNA samples. Hydroxymethylation in genes such as GATA4, GATA6, PROX1, ONECUT1, and MEIS2, which plays an important role in development and functioning of pancreas, as well as in genes YAP1, TEAD1, PROX1, IGF1, were found involved in cancer pathogenesis (78).

In addition, altered miRNA expression has also been associated with various cancers. miRNAs regulate gene expression at the transcriptional level. Significant upregulation of various miRNAs in PDAC serum samples includes, miR-215-5p, miR-122-5p, and miR-192-5p and decreased levels of miR-30b-5p and miR-320b, compared to chronic pancreatitis and hepatocellular carcinoma. Hence, this panel can serve as a non-invasive biomarker for an early detection of PDAC (79).

### 3.3 PDAC biomarkers

Proinflammatory cytokine IL-8, along with CA19-9 and carcinoembryonic antigen cell adhesion molecule 6 (CEACAM6), is considered a biomarker for PDAC diagnosis (80). Canonical markers cytokeratin 17 (CK17) for the BL subtype, GATA6 for the CLA subtype, and CK19 as a pan-cancer cell marker are also useful biomarkers (31). A serum biomarker panel of CA-125 and CA19-9 could be used for effective clinical management of PDAC patients undergoing neoadjuvant chemotherapy (NAC) (81). Yet another biomarker, CCL3 which is tumor derived, directs TGFβ signaling inducing macrophages to acquire its M2 phenotype and Lif secretion and sustaining a mesenchymal/basal-like subtype. TGFβ inhibition by galunisertib redirects macrophage polarization to M1, reducing Lif and shifting PDAC cells to a more epithelial/CLA phenotype, improving gemcitabine sensitivity (61). Mutant KRAS ctDNA is detected in patients at diagnosis, Neoadjuant chemotherapy and after resection. Clearance of ctDNA during NAC was associated with improved overall survival (OS). Detection of mutant KRAS G12V after NAC and resection was associated with shorter OS (82). Aberrant expression of microRNAs (miR) is known to be indicators of PDAC therapy resistance or therapeutic response (83). PDAC serum samples showed a significant overexpression of miR 215 5p, miR 122 5p, and miR 192 5p. On the other hand, PDAC had substantially lower levels of miR 30b 5p and miR 320b than hepatocellular carcinoma and chronic pancreatitis. This panel can therefore be used as a non-invasive biomarker for an early identification of PDAC (79).

Methylated DNA markers (FER1L4), along with CA 19-9, are also identified as blood-based biomarker for enhancing early detection and diagnostic sensitivity (84). Crucial biomarkers like CXCR4 and CD4 were identified which dysregulate the immune system, highlighting the significance of immune associated pathways (85). Differentially expressed genes were identified, mainly *PLAU* and *COL17A1* (86). Another set of differentially expressed genes which can act as biomarkers pertinent to PDAC includes CDK1, CDC20, KIF11, DLGAP5, CCNB1, BUB1, and CEP55 as hub genes which affect PDAC survival rate (87).

### 3.4 Epigenetic alterations- a potential driver for PDAC

**Figure 2:**
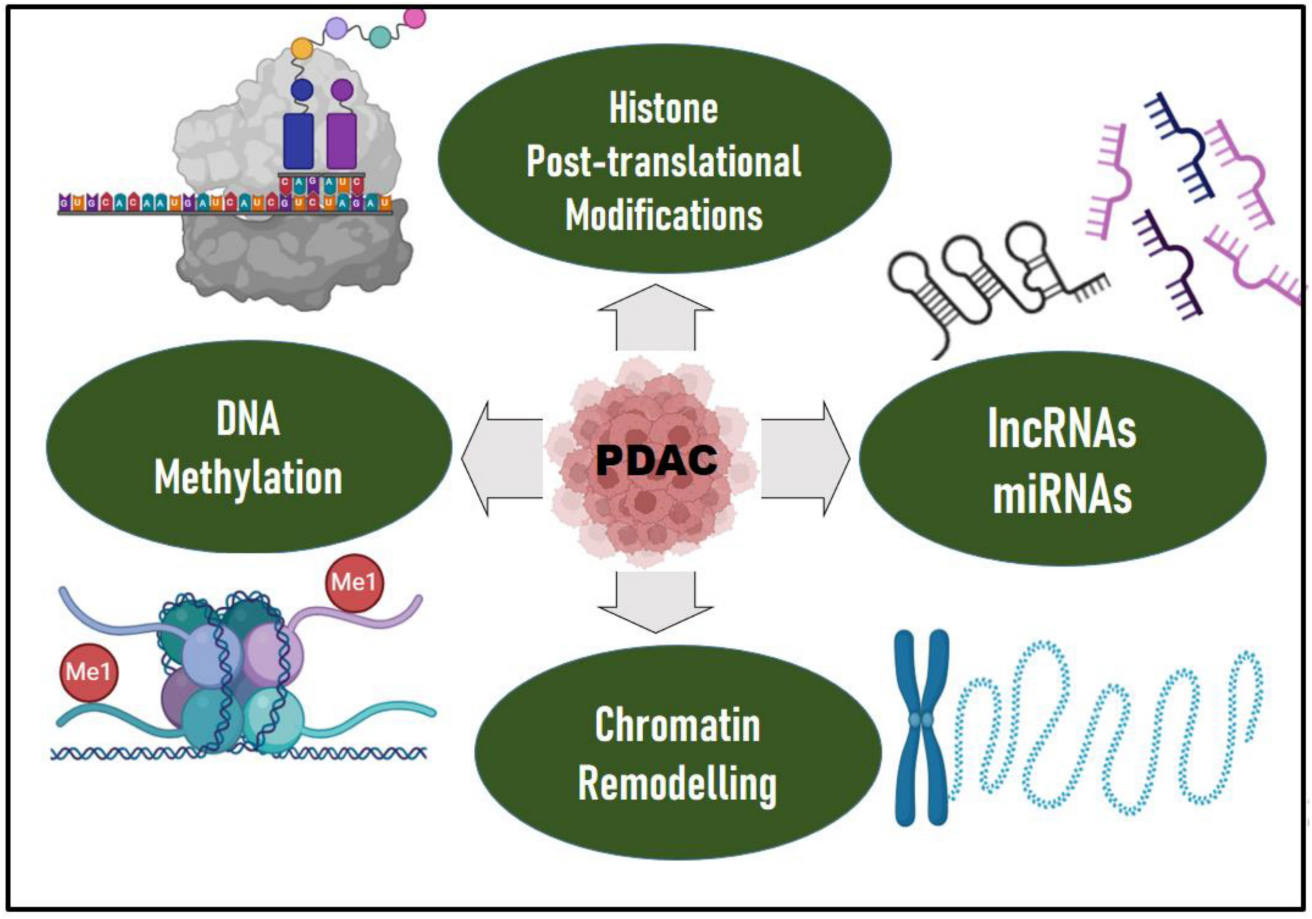
Epigenetic drivers of PDAC. Epigenetic mechanisms, including aberrant DNA methylation and histone modifications, chromatin remodeling, long non-coding RNAs (IncRNAs) and microRNAs (miRNAs), significantly contribute to inter- and intratumoral heterogeneity, disease progression and metastasis in PDAC. Epigenetic modifiers such as DNA methyltransferases, histone methyltransferases, histone acetyltransferases, histone demethylases, or deacetylases are mutated which contributes to PDAC. Mutations in epigenetic regulators such as TET2, DNMT3A, ASXL1, ARID1A/B, PBRM1, MLL2/3/4, KDM6A, SMARCA2/4 are evident in PDAC. Significant upregulation of various miRNAs such as miR-215-5p, miR-122-5p, and miR-192-5p and decreased levels of miR-30b-5p and miR-320b, were observed in PDAC compared to chronic pancreatitis and hepatocellular carcinoma. Protein arginine methyl transferase is also overexpressed in PDAC. The DKK1-Super Enhancer (DKK1-SE) in PDAC is characterized by aberrantly active histone modifications. Epigenetic modifications correlate with distinct PDAC subtypes in patient-derived xenografts, suggesting that distinct epigenetic states may underpin inter patient PDAC transcriptional heterogeneity.

Epigenetic mechanisms regulate gene transcription, and the proper functioning of these mechanisms is essential for normal development and tissue differentiation. Appropriate gene expression is maintained through self-regulatory mechanism that defines the epigenetic landscape and thus, any deviation or aberration can lead to cancer. The advancement in epigenomics has enriched our knowledge in understanding PDAC at the translational level. Evidences support that epigenetic alterations also contribute to the PDAC progression, a mechanism by which a genotype can have different phenotypic effects (88) (**Figure 2**). Although genetic mutations drive PDAC initiation, possibly triggering the early neoplastic lesions, they are it is not the only reason to explain its aggressive nature, as with tumour progression, the epigenomic landscape controls the gene expression directing the heterogenetic differentiation. Epigenetic mechanisms, including aberrant DNA methylation and histone modifications, and nucleosome remodeling significantly contribute to inter- and intratumoral heterogeneity, disease progression and metastasis (89). Epigenetic modifications correlate with distinct PDAC subtypes in patient-derived xenografts, suggesting that distinct epigenetic states may underpin inter patient PDAC transcriptional heterogeneity (90).

Epigenetic modifiers such as DNA methyltransferases, histone methyltransferases, histone acetyltransferases, histone demethylases, or deacetylases are mutated which also contributes to PDAC (91). A subset of PDAC patients have germline premature truncating variant mutations in epigenetic regulators (e.g., *TET2*, *DNMT3A*, and *ASXL1*) (92). In addition, whole exome and genome sequencing identified somatic mutations in the epigenetic regulators and chromatin remodeling complexes like ARID1A/B, PBRM1, MLL2/3/4, KDM6A, SMARCA2/4 (36, 37).

ATAC-seq (Assay for Transposase-Accessible Chromatin using sequencing) to analyze chromatin accessibility of surgically resected PDAC between patients found 1092 differentially accessible chromatin peaks. Subsequent computational TF motif analysis identified 61 TFs with binding motifs within these chromatin regions. These TFs included tumor-promoting ZKSCAN1 from the open chromatin regions of metastatic patients and tumor suppressor HNF1B from the open chromatin regions of non-metastases patients; and this can remodel chromatin landscape and accessibility to recruit or prevent TF binding as a mechanism to initiate tumor metastasis (93).

Histone methylation is one of the most complicated epigenetic modification process and arginine methyl transferase plays a major role. Arginine methyl transferase is overexpressed in PDAC. This induces expression of GLUT1 and HK2, which reduces the effectiveness of chemotherapeutic drugs (94). Furthermore, arginine methyl transferase inhibitors, in combination with chemotherapeutic drugs or immunotherapy, increased the anti-tumor effects by increasing CD8^+^ T cell count (94, 95).

Dickkopf-1 (DKK1) is increased in multiple tumors, and its silencing leads to tumor inhibition across diverse malignancies. The DKK1-Super Enhancer (DKK1-SE) in PDAC is characterized by aberrantly active histone modifications. Its principal component, enhancer e1, in conjunction with AP1 transcription factors JUND and FOSL2, triggers chromatin remodeling, hence facilitating the transcriptional activation of DKK1. The elimination of DKK1-SE significantly slows PDAC growth and alleviates the complexities of its microenvironment. DKK1-SE facilitated the advancement of PDAC by enhancing DKK1 expression, highlighting that the aberrant activation of DKK1 is induced by the epigenetic reprogramming of PDAC, thereby offering novel insights into the role of dysregulated histone modification in the progression of PDAC (96). Further understanding of the epigenetic landscape in PDAC could offer new potential biomarkers and tailored therapeutic approaches.

### 3.5 Long non-coding RNAs

About 25% of the RNA in human cells is made up of long non-coding RNAs (lncRNAs); PDAC carcinogenesis is frequently linked to their dysregulation (97). They modulate transcriptional and post-transcriptional gene expression, chromatin remodeling and epigenetic regulation (98). It is also reported to be involved in various steps of pancreatic cancer development and have a potential value in the diagnosis, treatment and prognostic prediction of PDAC (99). lncRNAs are highly upregulated in liver cancer (HULC) and Plasmacytoma variant translocation1 (PVT1) levels in tissue or plasma corelate with PDAC progression and clinical outcomes. lncRNAs that are upregulated in PDAC include HOX Transcript Antisense Intergenic RNA (HOTAIR), metastasis-associated lung adenocarcinoma transcript 1 (MALAT1), and H19 (100, 101).

### 3.6 Pathological conditions leading to PDAC

The microbiome has emerged as a contributor to oncogenesis in several gastrointestinal tract malignancies including pancreatic cancer. The dysbiosis of the microbial populations leads to inflammatory reactions and influences the immunological response of the host, therefore promoting cancer growth, as well as influencing the efficacy of anti-cancer treatment. Infection with *E. faecalis* may be involved in chronic pancreatitis progression, ultimately leading to development of pancreatic cancer (102). Although studies on the influence of bacteria on pancreatic cancer are still in their early years, it is clear that gut and oral microbiomes can act as potential pancreatic cancer diagnostic biomarkers and therapeutic targets (103). Pancreatic cancer immunogenicity increases with gut microbiota removal, which polarizes CD4^+^ T cells to Th1 and increased CD8^+^ T cell infiltration while decreasing MDSC infiltration (104). There is yet another complex relationship between Diabetes and PDAC (105). Long standing Type 2 Diabetes Mellitus is a known risk factor for PDAC (106).

## 4 Tumor immune microenvironment

The pancreatic TiME is indispensable for pancreatic cancer progression. It is a highly immunosuppressive environment, characterized by desmoplastic reaction with abundant stromal response. Reciprocal communication between cancer and stromal cells induces changes in cellular components of the PDAC TME, which can prime the primary tumor for metastasis and cell migration. The cells comprise of variable numbers of infiltrating immunosuppressive cells, such as tumor-associated macrophages (TAMs), myeloid-derived suppressor cells (MDSCs), regulatory T cells (T_reg_ cells), dysfunctional T cells and distinct cancer-associated fibroblast (CAF) subtypes, tumor-associated neutrophils, together with heterogenous extracellular matrix (ECM) impact patient prognosis and therapeutic outcome (6).

### 4.1 Immunosuppressive TME in PDAC

PDAC is characterized by the immunosuppressive TME with complex and diverse tumor infiltrating immune suppressive stromal, ECM and malignant tumor cells that contribute to poor prognosis and immunotherapy resistance. Several immunosuppressive cells are predominant in the TME that includes MDSCs, Tregs, and TAMs.

#### 4.1.1 Myeloid-derived suppressor cells

MDSCs are the heterogenous population of immature myeloid cells (CD15^+^, CD11b^+^), which have a significant role in immunosuppression (107). Immunosuppression mediated by MDSCs takes place via multiple signaling pathways. MDSCs suppress the anti-tumor functions of T cells and NK cells which leads to dismal prognosis; IL-6 is identified as a regulator of MDSC accumulation (108). Immunosuppression and immune evasion by MDSCs are also mediated by epidermal growth factor receptor- mitogen-activated protein kinases (EGFR-MAPK) - dependent upregulation of PD-L1 expression on tumor cells (109). It suppresses the anti-tumor immune functions of CD8^+^ T cells. Three major subsets of MDSCs have been identified in cancer and other chronic inflammatory conditions are: polymorphonuclear (PMN-MDSC), granulocytic (Gr-MDSC) and monocytic (M-MDSC) (110). Due to the immature nature of MDSCs, yet another phenotype, early-stage MDSC (eMDSC) has been proposed MDSC (110). Though they are not phenotypically different, they have distinct functional properties. M-MDSCs are typically differentiated by the surface markers CD11b^+^Ly6G^−^Ly6C^hi^ and PMN-MDSC with CD11b^+^Ly6G^+^Ly6C^lo^.

In PDAC patients, the amount of MDSCs in systemic circulation and bone marrow can be corelated with the stage of the disease (111). GM-CSF is one of the several growth factors implicated in this increase which is produced by malignant pancreatic epithelial cells (112). GM-CSF is a regulator of inflammation and immune suppression.

#### 4.1.2 Tumor associated macrophages

Resident macrophages differentiate into TAMs which have two phenotypes, M1 and M2; M1 express IL-1β and TNF-α (proinflammatory) and M2 phenotype is CD163^+^ and IL-10 producer (anti-inflammatory). Monocytes that infiltrate tumors exhibit phenotypes that share characteristics of M1 and M2; in the early stages of cancer, M1 phenotype predominates. As the disease progresses, they exhibit more of an anti-inflammatory M2 phenotype, leading to tumor evasion, which correlates with poor prognosis in PDAC patients. Both TAMs and MDSCs are known to promote pancreatic cancer stem cells (CSC) by the release of proinflammatory cytokines via STAT3 and NF-κB signaling which enhances CSC proliferation. MDSCs are known to differentiate into M2 macrophages in the PDAC TME under hypoxic conditions mediated by HIF-1α (113, 114).

HOXA9, a transcription factor, can potentiate PDAC progression by stimulating CD163 expression on TAM (115), which further increases therapy resistance (116). Twist is yet another transcription factor that contributes to tumor progression by epithelial -to- mesenchymal transition (EMT) and immunosuppression by reducing CD8^+^T and NK cells in the TME (117).

#### 4.1.3 Regulatory T cells

Tregs are the major players of immune suppression in PDAC TME. Treg modulation, in conjunction with other chemotherapy regimen, has emerged as a novel method for PDAC therapy. MDSCs also influence the production of Tregs, which have important role in immunotolerance (118). They are identified by forkhead box protein 3 (FOXP3) protein expression and high levels of IL-2 receptor alpha chain, CD25. Tregs bind to DCs and prevent them from activating CD8^+^T cells. Alternatively, TGF-β promotes the proliferation of Tregs, inhibiting antigen presentation by DCs (119, 120). Tregs inhibit the immune response against PDAC cells facilitating the premalignant stage to invasive PDAC (121), and thus, leading to higher mortality rates (122).

#### 4.1.4 Tumor associated neutrophils

Tumor associated neutrophils (TANs) represent an important cell population in PDAC TME, displaying extensive plasticity between anti-tumorigenic N1 and pro-tumorigenic N2 neutrophils in the TME, and is contributing to survival and immune infiltrations. Significantly increased N2 neutrophils favor tumor and is associated with poor survival. OS and recurrence free survival are associated with high infiltration of N1 neutrophils (123). N2 neutrophils improve angiogenesis via the secretion of matrix metalloproteinase and VEGF (124). They also promote metastasis by enhancing the expression of Bv8, S100A8, and S100A9 (premetastatic proteins). TANs secrete chemokine ligand 5 (CCL5) in abundance, and upregulate nectin2, subsequently enhancing cancer cell migration and invasion (125) by inhibiting the cytotoxic activity of CD8^+^ T cells. Secreted CCL5 and CCL17 also attract Tregs to TME that promote tumor progression (126). TGF-β, which is abundant in PDAC TME, also has a role in neutrophil polarization; blocking TGF-β improved antitumor TANs (127).

#### 4.1.5 Cancer associated fibroblasts restricting tumor infiltrating immune cells

Cancer associated fibroblasts (CAFs) are a major non-neoplastic, cellular constituent of the desmoplastic stroma in PDAC and can be derived from pancreatic stellate cells (PSCs) (128), tissue-resident fibroblasts, and tumor-infiltrating mesenchymal stem cells (MSCs) (129). CAFs are characterized by fibroblast activation protein (FAP) and α-smooth muscle actin (α-SMA), fibroblast-specific protein 1 (FSP1), and platelet-derived growth factor receptors alpha/beta (PDGFRα/β). In PDAC, CAFs are heterogenous, constituting myofibroblastic CAFs (myCAFs), inflammatory CAFs (iCAFs) and antigen-presenting CAFs (apCAFs). myCAFs and iCAFS switch between phenotypes based on the context (130). Fibrillar collagens, fibronectin, elastin, laminins and hyaluronan are ECM components in PDAC, which is secreted by myCAFs (131). They are the source of ECM and provide nutrients to tumor cells and make them more aggressive. Furthermore, they modulate cancer progression by various cytokines/chemokines like TGF-β, VEGF, IL-6, CXCL12. CAF migration and differentiation, mediated by ARP2/3, have been implicated in the initiation of PDAC (132). Immune escape of CAF’s mediated by releasing immunosuppressive cytokines and chemokines, such as IL-6, IL-1β, CXCL1, CXCL2, and CXCL12, and expressing immune checkpoint ligands (133).

ECM secretion by CAFs also builds the mechanical barrier and interstitial pressure in the TME that impedes drug delivery and immune cell infiltration leading to hypoxic TME. To ameliorate the hydrostatic pressure, hyaluronidase was used to breakdown hyaluronan in an attempt to improve drug delivery and tumor response (134, 135). A phase II trial of PEGylated hyaluronidase (PEGPH20), in combination with chemotherapy (gemcitabine/nab-paclitaxel), achieved 40% response, and the progression free survival (PFS) was 6.0 months (136).

Sonic hedgehog (Shh) signaling is overactive in PDAC. This overexpression leads to desmoplastic reaction (137, 138). Shh is a soluble ligand expressed by neoplastic cells in PDAC, which drives formation of a fibroblast-rich desmoplastic stroma (137, 139). By deleting the Shh in a PDAC mouse model, the stromal content was reduced by restraining tumor angiogenesis.

Mast cells in the TME promote *in vivo* growth of PDAC (140). Mast cell-derived factors like IL-13 and tryptase stimulate PSC proliferation leading to production of TGF-β2 and Smad2 phosphorylation (141). This results in desmoplastic stroma which promotes proliferation of PSCs and eventually leading to poor prognosis.

#### 4.1.6 Cytokines and chemokines

Chemokines are key drivers of inflammation, and the major promoters of cancers. However, a combination of chemokines (CCL19, CCL21, CXCL9, CXCL10, CXCL12 and XCL1) together with cytokines IL-2, IL-12, granulocyte–macrophage colony-stimulating factor (GM-CSF), stimulate T cells, NK cells or tumor antigen-pulsed DCs (142, 143) contributing to TME. Therefore, targeting chemokines or chemokine receptors is a promising strategy for enhancing immunotherapy in PDAC.

Role of CCL2 in M-MDSC migration into tumor site and accumulation occurs via CCR 2, 4, and 5 (144). CXCR2 is a chemokine receptor that mediates MDSC recruitment to the PDAC TiME. CXCR2^+^ MDSCs migrate in response to ligands like CXCL1, CXCL2, and CXCL5, which are often secreted by PDAC cells. Targeting CXCR2 has shown potential in reducing MDSC infiltration and enhancing antitumor immunity.

#### 4.1.7 Soluble factors

There are many soluble mediators that play a significant role in the PDAC immune response. Properdin, the only known positive regulator of the alternative complement pathway, is significantly elevated in the early stages of PDAC but declines in advanced disease. Neutrophils, which store properdin in their granules, contribute to its increased expression in patients, with high neutrophil infiltration correlating with the CLA PDAC subtype and improved survival outcomes (145). Properdin exhibits anti-tumorigenic properties by promoting apoptosis in BL PDAC cell lines. However, its levels are markedly reduced in the blood of PDAC patients, suggesting that complement suppression aids immune evasion and reduces the efficacy of cancer immunotherapy. Human surfactant protein D (SP-D) is another soluble factor that modulates cytokines and chemokines. Kaur et al. studied the role of SP-D in suppressing EMT in PDAC by downregulating TGF-β signaling (146). A recombinant fragment of human SP-D, containing homotrimeric neck and C-type lectin domains, has been shown to induce apoptosis in pancreatic cancer cell lines through the Fas-mediated pathway (147). These studies suggest that soluble factors can potentially be used to therapeutically target pancreatic cancer cells. Another complement component, C1q, plays a pro-metastatic role in PDAC, particularly in hepatic metastases. C1q expression progressively increases from normal pancreatic tissue to primary tumors and then to hepatic metastases driven by M2 macrophages, which are known for their tumor-promoting and immunosuppressive functions. C1q facilitates PDAC cell migration and invasion, contributing to disease progression. Additionally, alterations in the TME, including upregulation of the complement cascade, have been linked to enhanced metastatic potential in PDAC (148). Recent spatial transcriptomic study uncovered the role of C1q in contributing to the establishment of the immune-suppressive microenvironment that supports tumor progression. The role of C1q in promoting metastasis, especially in liver metastases, is critical as it interacts with immune cells, particularly macrophages, influencing the tumor’s ability to thrive in new locations. This insight supports the idea that complement subcomponents such as C1q can aid in tumor adaptation to the metastatic niche, promoting PDAC spread (149).

### 4.2 Aberrant signaling pathways in PDAC

Aberrant cell signaling is an important hallmark of cancer progression. Cancer cells as well as the cells in the stromal microenvironment continuously interact and perceived by cellular signaling networks. Signaling pathways that drive tumor progression and therapy resistance include KRAS, TGF-β, Notch, hypoxia-inducible factor (HIF), and Wnt/β-catenin. Aberrant signaling results from multiple genetic and epigenetic alterations such as the mutation in driver genes (KRAS, *CDKN2A*, *p53*), genomic amplification of regulatory genes (*MYC*, *IGF2BP2, ROIK3*), deregulation of chromatin-modifying proteins (HDAC, WDR5), among others. Pancreatic cancer cells need continuous K-Ras signaling for their proliferation and survival. Inactivation of GTPase due to mutation constitutively activates Ras signaling and downstream effector pathways (150). The downstream effectors in pancreatic cancer predominantly act through canonical Raf/MAPK/extracellular signal-regulated kinase (Erk), PI3Ks/(PDK-1)/Akt, RalGEFs, and phospholipase Cϵ (150). The most recurrently mutated signal transduction pathway in PDAC is TGFβ signaling, thus, inhibition of this pathway can lead to therapeutic approach (60, 151).

### 4.3 Microbiome influence on chemotherapy resistance

Systemic therapy in PDAC patients eventually leads to drug resistance and there is evidence to suggest that microbiota have potential to induce chemotherapy resistance (152). Intratumor bacteria are found in many cancers including PDAC (153). The most common bacteria in the PDAC intratumor microbiome are *Gamma-proteobacteria*, with the dominant genus *Pseudomonas* (104), which carries long-form cytidine deaminase that metabolizes the chemotherapeutic drug gemcitabine (2′,2′-difluorodeoxycytidine), into its inactive form (2′,2′-difluorodeoxyuridine). The anti-cancer efficacy of gemcitabine is negatively affected by the cytidine deaminase activity of *Mycoplasma*, which leads to drug catabolism in the TME (154). This deamination is further potentiated by mycoplasma-derived pyrimidine nucleoside phosphorylase (PyNP) activity. Intratumoral microbiota of BL PDAC are A*cinetobacter*, *Pseudomonas*, and *Sphingopyxis*, which are associated with worse prognosis due to induction of inflammation (155). Microbial dysbiosis is another reason for gemcitabine and albumin-bound paclitaxel resistance (156).

### 4.4 Metabolic regulation of PDAC

Tumor tissues have lower levels of glucose, high glycolytic intermediates, creatine phosphate, and the amino acids glutamine and serine, which are the main metabolic substrates, according to metabolomic comparisons between human PDAC tumor tissue and normal surrounding tissue (157). Acetyl-CoA, which is produced from acetate, is linked to protein acetylation. These regulatorty processes include altered cellular signaling, epigenetic alterations, gene expression, DNA replication, and DNA damage repair. Immunohistochemical (IHC) examination of pancreatic cancer samples reveals increased histone acetylation. Another defining feature of tumor growth is acidosis, which may result from an overactive glycolytic metabolism. Acidosis affects tumor metabolism by increasing mitochondrial activity and decreasing glycolysis (158). Targeting the metabolic nodes is another mode for improving therapy in PDAC.

## 5 Intratumor heterogeneity

Intratumor heterogeneity is another major hurdle for effective therapeutic options. Heterogeneity is observed in CSCs, transcriptional and epigenetic variation, epithelial mesenchymal transition and metabolic difference (159). Tumor initiating capacity or the stemness of PDAC may be attributed to the presence of CSCs in cancer cell populations. It is identified by various cell surface markers CD133, CXCR4, CD44, CD24 and EpCAM and several other markers (160, 161). Yet another marker is cell surface tetraspanin, CD9, for both murine and human PDAC CSCs (162). mRNA binding proteins, Msi1 and Msi2, have been characterized in murine PDAC having tumor initiating capacity. Furthermore, Msi2 directly binds and modulates the transcript levels of epigenetic modifiers such as Brd4 and Hmga2 (163). Different therapeutic strategies have been developed to target CSCs (164).

Transcriptional and epigenetic control indicate a great ability of PDAC neoplastic cells to modify their phenotypic identity. Epigenetic co-regulators and lineage-specific TFs are important indicators of PDAC subtype specificity and disease progression. The function of AP1 TFs in the heterogeneity of PDAC has recently been reported, emphasizing the divergent tumor states driven by JUNB/AP1 and cJUN/AP1. JUNB preserves a CLA phenotype by inhibiting inflammatory signals and stabilizing differentiation factors such as GATA6, while cJUN fosters a BL phenotype through TNF-α-mediated macrophage recruitment (165). The absence of JUNB results in an inflammatory milieu, diminishing T cell infiltration and facilitating BL transition. Targeting TNF-α by immunotherapy and chemotherapy increases T cell presence, inhibits macrophages, and enhances survival in mice, indicating a potential therapeutic approach to mitigate PDAC immune suppression and aggressiveness (165).

### 5.1 Stromal heterogeneity

Stromal microenvironment influences the intratumoral composition of PDAC (166). Normal and activated stromal subtypes are identified according to the PDAC subtype classification (22), the latter having worse outcomes. Patients with activated stroma have higher myCAF and csCAF myofibroblastic and immunogenic fibroblasts, M2 macrophages and Tregs. Normal stroma has higher PSCs (167).

## 6 Therapeutic strategies in PDAC

**Figure 3:**
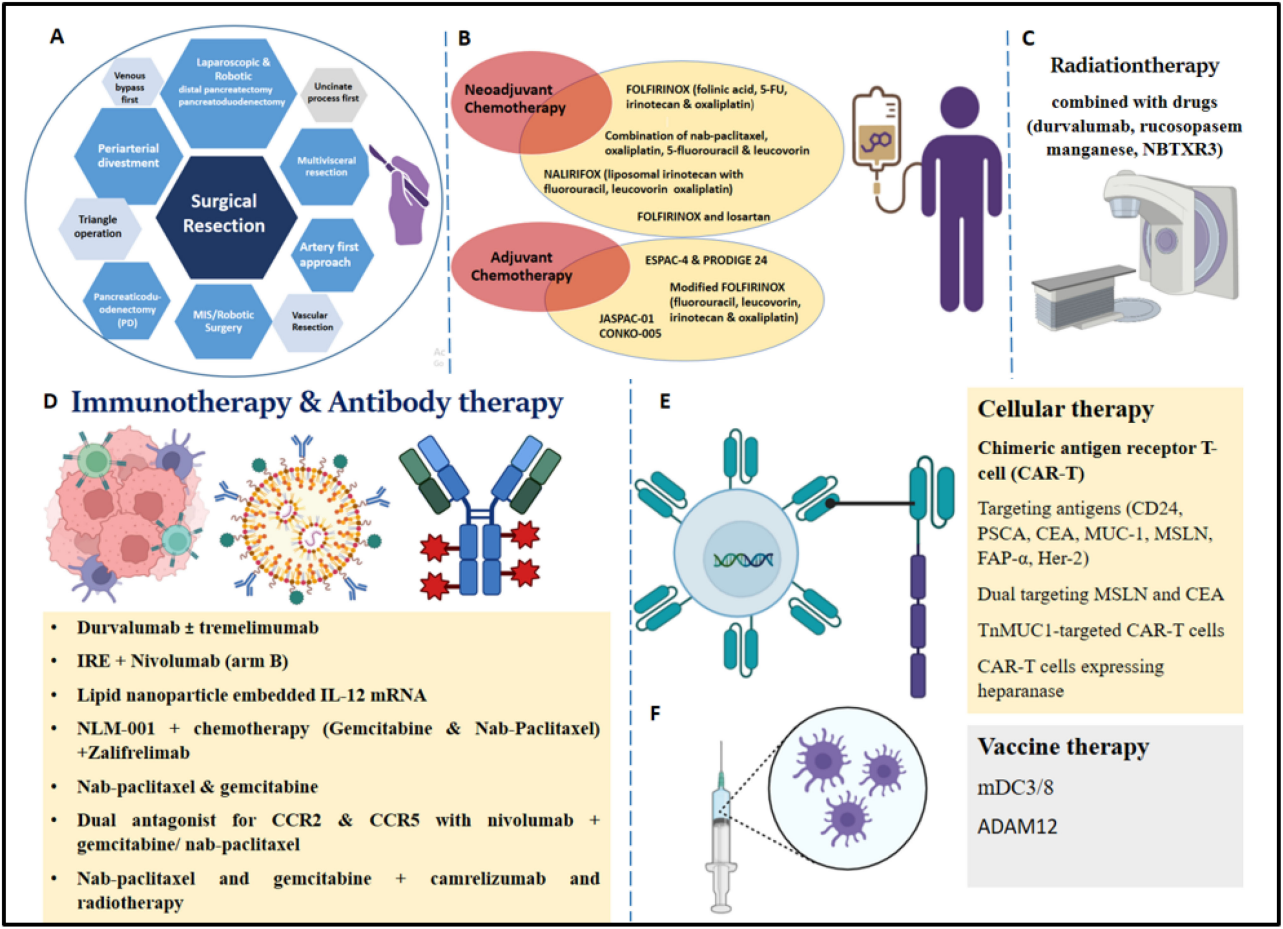
Various therapeutic options for treatment of PDAC patients. **(A)** Surgical resection (a. Pancreaticoduodenectomy, b. artery first approach, uncinate process first, triangle operation, c. venous bypass first, d. periarterial divestment, vascular resection, multivisceral resection, f. MIS/Robotic Surgery, g. laparoscopic and robotic distal pancreatectomy/robotic pancreatoduodenectomy. **(B)** Neoadjuvant Chemotherapy (a. FOLFIRINOX regimen stage IV disease, b. novel combination of nab-paclitaxel, c. oxaliplatin, 5-fluorouracil, and leucovorin in advanced PDAC patients, d. phase III NALIRIFOX trial, e. FOLFIRINOX and losartan); Adjuvant chemotherapy (a. ESPAC-4 and PRODIGE 24, b. modified FOLFIRINOX regimen, c. JASPAC-01, d. CONKO-005, e. combination of nab-paclitaxel and gemcitabine). **(C)** Radiotherapy in combination with different drugs (durvalumab, rucosopasem manganese, and NBTXR3). **(D)** Immunotherapy (a. durvalumab with or without tremelimumab, b. IRE (Irreversible Electroporation) + Nivolumab (arm B), c. Lipid nanoparticle embedded IL-12 mRNA, d. NLM-001 + chemotherapy (Gemcitabine and Nab-Paclitaxel) +Zalifrelimab; Antibody therapy (a. durvalumab with tremelimumab, b. nab-paclitaxel and gemcitabine, c. dual antagonist for CCR2 and CCR5 with nivolumab and gemcitabine/ nab-paclitaxel, d. nab-paclitaxel and gemcitabine + camrelizumab and radiotherapy. **(E)** Cellular therapy (a. chimeric antigen receptor T-cell (CAR-T), b. targeting antigens (CD24, PSCA, CEA, MUC-1, MSLN, FAP-α, Her-2), c. dual targeting MSLN and CEA, d. TnMUC1-targeted CAR-T cells, e. CAR-T cells expressing heparanase. **(F)** Vaccine therapy (a. dendritic cell vaccine (mDC3/8), b. vaccination against ADAM12).

Chemotherapy profoundly alters the PDAC TME and might lead to further resistance to immunotherapy due to reduced inhibitory check point molecule expression and interactions involving CD8^+^T cells (31). As it tends to develop therapy resistance and has such a worse prognosis, PDAC is a malignant tumor with a very high mortality rate. Although chemotherapy, radiation therapy and immunotherapy are standard treatments, systemic chemotherapy is still the most used approach. However, immune checkpoint inhibitors, which have had considerable success in treating other solid tumor types show limited effectiveness in PDAC. A key feature of PDAC is its inherent resistance to drug therapy. This inherent chemoresistance arises from various cellular mechanisms, including drug efflux, stemness properties, cell cycle regulation, and an elevated apoptotic threshold in response to drug exposure. These resistance mechanisms are driven by multiple oncogenic signaling pathways and dysregulated cellular processes (168). The various therapeutic options in PDAC are schematically represented in **Figure 3**.

### 6.1 Surgical resection

Surgery is considered as the primary treatment option for PDAC, along with adjuvant. Since 1940, Pancreaticoduodenectomy (PD) has been widely applied; Whipple reported the classical procedure including distal gastrectomy and total duodenectomy and this has been modified (169). Specific techniques used in the surgery are as follows: artery first approach (170), uncinate process first (171), triangle operation (172, 173), venous bypass first (174–176), periarterial divestment (177, 178), Vascular (Venous and Arterial) Resection (179), multivisceral resection (180, 181), MIS/Robotic Surgery (182–184), laparoscopic and robotic distal pancreatectomy (185, 186), and laparoscopic and robotic pancreatoduodenectomy (187, 188). Over years, pancreatic surgical procedures have improved leading to 5% reduction in mortality (189).

### 6.2 Chemotherapy

Chemotherapy is the primary therapeutic option for locally progressed, metastatic, or recurrent PDAC. A combination of nab-paclitaxel and gemcitabine has been approved as a first-line therapy for locally advanced unresectable or metastatic PDAC due to a higher median OS rate than gemcitabine alone (190).

#### 6.2.1 Neoadjuvant chemotherapy

Neoadjuvant chemotherapy, refers to chemotherapy administered before primary treatment with surgery and/or radiation therapy (191). A phase III randomized trial of gemcitabine alone versus gemcitabine plus capecitabine in metastatic PDAC patients demonstrated improved objective response rate and progression-free survival (192). The FOLFIRINOX regimen (folinic acid, 5-FU, irinotecan, and oxaliplatin) has shown a 31.6% response rate in patients with stage IV disease, although with increased hematologic and neurologic toxicity (193).

A phase III trial investigated the safety and efficacy of nab-paclitaxel plus gemcitabine versus gemcitabine monotherapy in metastatic PDAC. Despite increased rates of peripheral neuropathy and myelosuppression, the combination therapy improved OS, progression-free survival, and response rate (190). A phase I trial confirmed the improved safety and tolerability profile of nab-paclitaxel plus gemcitabine, along with considerable anti-tumor activity (194).

A study found that drug funding decisions led to increased uptake of newer treatments and improved survival in advanced pancreatic cancer patients receiving first-line palliative chemotherapy. Both FOLFIRINOX and gemcitabine/nab-paclitaxel demonstrated survival benefits in specific patient populations (195).

A more recent study evaluated the novel combination of nab-paclitaxel, oxaliplatin, 5-fluorouracil, and leucovorin in advanced PDAC patients, showing improved activity and potential as an alternative to FOLFIRINOX with less gastrointestinal toxicity (196). The phase III NALIRIFOX trial (liposomal irinotecan with fluorouracil, leucovorin, and oxaliplatin) versus nab-paclitaxel and gemcitabine in metastatic PDAC patients showed improvements in overall and progression-free survival (197).

For patients who are receiving extended neoadjuvant FOLFIRINOX followed by surgical resection, survival outcomes increase by reducing the tumor for better access to surgery (198). Yet another study targeting the desmoplastic stroma on locally advanced PDAC showed better response with neoadjuvant FOLFIRINOX followed by chemoradiotherapy (CRT) and then surgical attempt. Losartan was included in this study as it could inhibit the renin-angiotensin system signaling that improved the delivery of chemotherapy to the tumor and inhibited collagen I synthesis (199, 200). Treatment with FOLFIRINOX and losartan was associated with a significant reduction in plasma thrombospondin-1 and TGF-β levels.

#### 6.2.2 Adjuvant chemotherapy

Adjuvant therapy is given to PDAC patients as a curative therapy after surgery. The current standard of treatment has been established by the ESPAC-4 and PRODIGE 24 studies, even though single-agent adjuvant therapy with gemcitabine or 5FU has demonstrated survival advances. In 732 patients, ESPAC-4 indicated that gemcitabine/capecitabine increased OS compared to gemcitabine alone. In contrast to gemcitabine in the adjuvant context, modified FOLFIRINOX (lower irinotecan and no bolus 5-FU) also improved OS, according to PRODIGE 24, a major global phase 3 study (193, 201). Adjuvant therapy with a modified FOLFIRINOX regimen (fluorouracil, leucovorin, irinotecan, and oxaliplatin) led to significantly longer survival than gemcitabine among patients with resected pancreatic cancer, at the expense of a higher incidence of toxic effects (202). APACT phase III trial that used adjuvant nab-paclitaxel and gemcitabine in comparison with gemcitabine alone showed better OS with combination therapy (203). The PREOPANC-3 trial is a randomized, multi-center, phase III trial; patients were either given 8 cycles of mFOLFIRINOX before surgery and 4 cycles after (arm 1), or surgery followed by 12 cycles of mFOLFIRINOX (arm 2) (204).

Earlier clinical trials using JASPAC-01, where S1, an oral drug containing tegafur (prodrug of flurouracil), gimeracil (inhibitor of dihydropyrimidine dehydrogenase), and oteracil potassium (reduce gastrointestinal toxicity) were recognized to be a standard adjuvant chemotherapy for patients with resected PDAC compared to adjuvant gemcitabine (205). Yet another trial, CONKO-005, involved adjuvant chemotherapy with targeted therapy using Gemcitabine and Erlotinib in comparison with Gemcitabine monotherapy in patients after R0 (resection for cure or complete remission) resection of PDAC which did not provide an improved survival benefits (206).

### 6.3 Radiation

Although there is little evidence that adjuvant radiation therapy works, it is widely used. The first randomized trial was the GITSG study in 1985, which examined 43 PDAC patients with negative surgical margins and compared adjuvant chemoradiation (40 Gy split-course with weekly 5-FU) (207). The original trial was prematurely ended due to inadequate patient enrolment (intended target was 100 patients), although it showed an OS benefit with CRT (median survival of 20 months versus 11 months). Even though CRT subsequently became the accepted adjuvant treatment, its benefit for survival has been questioned by various randomized trials (208, 209). Hepatic artery infusion chemotherapy may help avoid hepatic metastases and enhance long-term survival following a radical pancreatectomy for PDAC. However, considering the current investigation was retrospective and involved only a limited number of individuals, a larger, prospective study is required to verify these findings (210). Furthermore, with the development of new systemic therapies and a greater emphasis on managing local disease, radiation therapy is probably going to become even more significant (211). Current research is making use of radiation therapy combined with different drugs for locally advanced PDAC. Some of the drugs that are being tested include durvalumab, a PD-L1 inhibitor; rucosopasem manganese, which mimics superoxide dismutase; and NBTXR3, a radio-enhancer (212, 213). Whether neoadjuvant chemoradiation will improve OS in PDAC is difficult to predict (214).

### 6.4 Immunotherapy

Vaccines, adoptive cell therapies, and checkpoint inhibitors are all examples of cancer immunotherapies. These are designed to alter the activation of co-inhibitory and co-stimulatory receptors on immune cells, particularly T cells, which are critical for modulating adaptive immunological responses. To increase T cell activity against PDAC, clinical trials have assessed the use of immune checkpoint inhibitors that target the cytotoxic T lymphocyte antigen-4 (CTLA-4) and programmed cell death-1 (PD-1/PD-L1 pathways). PD-1/PD-L1 inhibition regulates the anti-tumor T cell response in peripheral tissues, while CTLA-4 blockade impacts Treg cell activity and enhances immunity against tumors. Blocking immunotherapy interferes with the interaction between the PD-1 receptor on T-cells and its ligand, PD-L1, which is often expressed on tumor cells. This interaction affects T cell action that normally blocks them from effectively targeting cancer. However, using this therapy, specific antibodies to block this interaction between natural immunity against tumor tissue is boosted (215).

A phase II randomized trial used durvalumab with or without tremelimumab, in 65 patients with previously treated metastatic PDAC. In both the combination therapy (3.1%) and the monotherapy (0%), the objective response rate were extremely low. It was challenging to determine whether treatment response and PD-L1 expression or microsatellite instability status were related due to the small number of patients recruited in the study (216).

A randomized phase I trial to study the safety of the combination therapies IRE (Irreversible Electroporation) + Nivolumab (arm B) targeting the PD-1 receptor on T-cells and CpG (Toll-Like Receptor 9) ligand stimulated dendritic cells to release type I IFN, activated NK and infiltrating CD8+T cells and created a more pro-immunogenic TME. More studies should be done to prove the safety and clinical reproducibility (217).

Novel therapeutic strategies for the reversal of T cell exhaustion is a good treatment option. IL-12, a potent proinflammatory cytokine, enhances T cell effector function. Lipid nanoparticle embedded IL-12 mRNA utilized for the intratumoral delivery has proven safe and tolerable (NCT03946800) along with stereotactic body radiation therapy for the optimal activity which resulted in long term OS (218).

Another study utilized two prognostic genes to categorize M2-like TAMs in PDAC into anti-tumor bM2-like TAMs and pro-tumor mM2-like TAMs. The bM2-like TAMs activate T lymphocytes via ALCAM/CD6 and produce prognosis-favorable αSMA+ myofibroblasts by secreting TGFβ, providing insights into TAM heterogeneity, prognosis prediction, and immunotherapy for PDAC (219). SLC12A5 is an integral membrane potassium-chloride cotransporter mainly involved in maintaining chloride homeostasis in neurons, is a potential prognostic biomarker for human cancer (220). ENPP2 (ATX) encodes a secreted enzyme that functions as both a phosphodiesterase and a phospholipase. Higher expression of these genes was correlated with improved survival in PDAC patients. This provides a new target for immunotherapy in PDAC.

Another ongoing phase Ib/IIa trial examined safety and efficacy of NLM-001, the Hedgehog inhibitor and chemotherapy (Gemcitabine and Nab-Paclitaxel) +Zalifrelimab, CTLA-4 blocker within the 17 months’ time frame, the outcome is yet to be reported (221).

#### 6.4.1 Antibody therapy

The most used approach for immunomodulation is monoclonal antibodies (222); targeting PD-1/PD-L1 and CTLA-4 has proven highly effective across numerous solid tumors. Combination of antibody and chemotherapy targets the tumor specific receptors such as anti-EGFR/VEGF antibody for increased improvement in tumorigenesis (223). A study that added cetuximab to the standard therapy showed no significant change in OS rate and toxicity (224). A phase II clinical trial tested durvalumab (anti-PD-L1 monoclonal antibody) with tremelimumab (anti-CTLA-4 monoclonal antibody) versus durvalumab monotherapy in individuals, previously treated with chemotherapy for metastatic PDAC, did not increase OS rate (216). This highlights the need for translational studies that can circumvent the immunosuppressive and desmoplastic stroma of PDAC TME. Dual antagonist for CCR2 and CCR5 with nivolumab and gemcitabine/nab-paclitaxel in Borderline Resectable and Locally Advanced PDAC appeared to be safe and neoadjuvant use did not lead to delay in surgery (225).

A clinical trial of two years is being conducted to study the safety and efficacy of nab-paclitaxel and gemcitabine plus camrelizumab (Anti-PD-1 antibody) and radiotherapy versus nab-paclitaxel and gemcitabine monotherapy for locally advanced PDAC which showed super efficacy over the latter (226).

#### 6.4.2 Vaccine therapy

DCs are the potent APCs which stimulate helper T cells (227). In PDAC, an open label trial to determine effects of a DC vaccine (mDC3/8) (primer and booster) for resective PDAC has been carried out, but the results are not published yet (228). Precision based and immunotherapy based clinical trials will open therapeutic options such as PARP inhibitors and PD-1 blockade for well defined subtypes of PDAC (229).

Targeting the desmoplastic stroma is an essential step for an effective therapy in PDAC. Disintegrin metalloprotease, ADAM12, expressed in CAFs and tumor cells, is reactivated in fibroblasts during fibrogenesis in desmoplasia. Vaccination against ADAM12 depletes CAFs and delays tumor growth, by reducing ADAM12^+^ CAFs, and decreases deposition of ECM. It also increases cytotoxic CD8 ^+ ^T cell response and re-localization of T cells within the tumor tissue. Furthermore, it induces vascular normalization with decreased tumor hypoxia. This study highlights the importance of immunotherapies based on immunization that target CAFs and tumor desmoplasia (230).

### 6.5 Cellular therapy

Chimeric antigen receptor T-cell (CAR-T) therapy uses a patient’s own T cells, which are collected and genetically modified to recognize and destroy cancer cells carrying a specific target antigen (231). CAR T-cell therapy in PDAC targets antigens such as CD24, Prostate stem cell antigen (PSCA), CEA, Mucin-1 (MUC-1), Mesothelin (MSLN), FAP-α, and human epidermal growth factor receptor 2 (Her-2) (232, 233). Dual targeting of cancer antigens in KRAS mutated PDAC, MSLN and CEA by computational approaches for elucidation of anti tumor response has been tested (234). Engineered CAR-T cells made to express heparanase (HPSE) showed improved capacity to degrade the ECM, which promoted tumor T cell infiltration and antitumor activity, a strategy to treat stroma-rich solid tumors (235). A recent phase I, open-label, multi-center, first-in-human trial of TnMUC1-targeted CAR-T cells in patients with advanced TnMUC1-positive solid tumors and multiple myeloma including PDAC is being conducted (236).

## 7 Strategies to overcome heterogeneity and future directions

Identifying the key oncogenic drivers can navigate PDAC therapeutics. This includes precision-based therapies against denoted KRAS mutation like G12D, G12V, G12R, G12C and other mutations (229). Since epigenetic changes drive much of the heterogeneity in PDAC, targeting these modifications can also be considered. Drugs that inhibit DNA methylation, histone modifications, and non-coding RNAs need to be investigated. Transcriptionally defined molecular subtype-based treatment regimen by gene expression profiling and immunotherapeutic approaches that can reprogram the TiME can revolutionize PDAC treatment. Another strategy could target immunosuppressive myeloid cells and reprogram DCs, macrophages and CAFs, leading to increased CD8^+^ T lymphocyte activity against PDAC cancer cells.

Efforts to enhance immune infiltration in PDAC TME have included targeting CXCR4, to increase T-cell chemotaxis. In fact, a combination of PD-1 and CXCR4 inhibition resulted in enhanced T-cell expansion and tumor cell death in pre-clinical models (237). CD40 activation may represent a strategy to reverse T-cell exhaustion, enhancing the anti-cancer effects of the TiME. Consistent with this notion, agonistic CD40 antibodies were shown to increase T-cell mediated cancer death and, in combination with chemotherapy, may rescue ICI sensitivity (238).

There is an insufficiency of preclinical pancreatic cancer models that replicate the extracellular, cellular, and biomechanical components of tumor tissues to evaluate responses to immunotherapy. To overcome this constraint and investigate the effects of immunotherapy in conjunction with chemotherapy, a multicellular 3D cancer model with a star-shaped poly (ethylene glycol)–heparin hydrogel matrix has been developed. Human pancreatic cancer cells, CAFs, and myeloid cells are cultured within hydrogels to replicate essential elements of tumor tissues, and cellular responses to therapy are evaluated. The combination of the CD11b agonist ADH-503 with anti-PD-1 immunotherapy and chemotherapy results in a substantial decrease in tumor cell viability, proliferation, metabolic activity, immunomodulation, and the release of immunosuppressive and tumor-promoting cytokines (239).

Using a combination of drugs that target different signaling pathways can help in overcoming resistance and heterogeneity. Multifaceted mechanisms are operating in the PDAC TME, influencing the response to chemotherapy, radiotherapy, and immunotherapy. Precision-based therapeutic decisions will help to overcome the heterogeneity in PDAC. Understanding the underlying reason to chemotherapy resistance can improve the efficiency of drugs. Advanced techniques like scRNA-seq and spatial transcriptomics can help in understanding the heterogeneity at a cellular level. This can lead to more personalized treatment approaches.

## Glossary

ACVR1: activin receptor type-1
ARIDIA: AT-rich interactive domain 1A
ARP2: Actin Related Protein
ASXL: Additional sex combs-like
ATM: ataxia telangiectasia mutated
ATAC-seq: Assay for Transposase-Accessible Chromatin sequencing
BEAMing: beads, emulsion, amplification, magnetics
BL: Basal like
BRCA: breast cancer
Brd: bromodomain
CA: carbohydrate antigen
CAF: cancer associated fibroblasts
CAR-T cell: Chimeric antigen receptor (CAR) T-cell
CCL: Chemokine (C-C motif) ligand
CCNB1: cyclin cyclin B1
CDC: Cell division cycle
CDKs: cyclin dependent kinase
CDKN2A: cyclin-dependent kinase inhibitor 2A
CDS: coding DNA sequence
CEP55: centrosomal protein 55
cfDNA: circulating cell free DNA
CK: Cytokeratin
CLA: classical
CNV: copy number variation
COL17A1: collagen type XVII alpha 1 chain
CRT: chemoradiotherapy
ctDNA: circulating tumor-specific cell-free DNA
CT: computerized tomography
CTLA-4: cytotoxic T-lymphocyte-associated protein 4
CSC: Cancer stem cell
CXCR4: CXC chemokine receptor 4
CXCL1: chemokine (C-X-C motif) ligand 1
DKK1: Dickkopf-1
DPC4: deleted in pancreatic cancer 4
DLGAP5: discs large-associated protein
DNMT3A: DNA methyltransferase 3A
ECM: extra cellular matrix
EGFR: epidermal growth factor receptor**;** eMDSC, early-stage myeloid-derived suppressor cells
EMT: epithelial-to-mesenchymal transition
EPC1: enhancer of polycomb homolog 1
EPCAM: Epithelial cell adhesion molecule
ENPP2: ectonucleotide pyrophosphatase/phosphodiesterase 2
ERBB2: erythroblastic leukemia viral oncogene homolog 2
FOXP3: forkhead box protein 3
FSP1: ferroptosis suppressor protein 1
Gr-MDSC: granulocytic myeloid-derived suppressor cells
HER2: human epidermal growth factor receptor 2
HMGA 2: high-mobility group A2
HPSE: Heparanase
HIF: hypoxia-inducible factor
HOTAIR: HOX Transcript Antisense Intergenic RNA
HK: hexokinase 2
HULC: highly upregulated liver cancer
IGF1: insulin-like growth factor 1
IHC: Immunohistochemistry
IL: Interleukin**;** IPMN, intraductal papillary-mucinous neoplasm
KRAS: Kirsten rat sarcoma viral oncogene homolog
KLF: Krüppel-like factor
Lif: leukemia inhibitory factor
lncRNA: long non coding RNA
MALAT1: metastasis-associated lung adenocarcinoma transcript 1
MAPK: mitogen activated protein kinase
MEIS2: Meis homeobox 2
MDM2: Murine double minute 2
MDSCs: myeloid-derived suppressor cells
MCN: mucinous cystic neoplasm
miR: micro RNA
MLL: Mixed-Lineage Leukemia
MRD: minimal residual disease
mRNA: messenger RNA
mtRNA: mitochondrial RNA
NALCN: Sodium leak channel, non-selective
NAC: Neoadjuvant chemotherapy
NF-KB: nuclear factor-kappa B
NK cells: Natural killer cells
PC: pancreatic cancer
PDAC: pancreatic ductal adenocarcinoma
PanIN: pancreatic intraepithelial neoplasia
PSC: pancreatic stellate cells
PVT1: plasmacytoma variant translocation 1
TAM: tumor associated macrophages
TAN: tumor associated neutrophils
TCGA: The cancer genome atlas
TGF-β: transforming growth factor beta**;** TME, tumor microenvironment
TNM: Tumor, Node, Metastasis
TiME: tumor immune microenvironment
T_reg_ cells: regulatory T cells
VEGF: Vascular endothelial growth factor
5mC: 5-Methylcytosine
5hmc: 5 hydroxymethyl cytosine

## References

[1] Kleeff J Korc M Apte M La Vecchia C Johnson CD Biankin AV. Pancreatic cancer. Nat Rev Dis Primers. (2016) 2:1–22. doi: 10.1038/nrdp.2016.24 27158978

[2] Sohn TA Yeo CJ Cameron JL Koniaris L Kaushal S Abrams RA. Resected adenocarcinoma of the pancreas—616 patients: Results, outcomes, and prognostic indicators. J Gastrointestinal Surg. (2000) 4:567–79. doi: 10.1016/S1091-255X(00)80105-5 11307091

[3] Fink DM Steele MM Hollingsworth MA. The lymphatic system and pancreatic cancer. Cancer Lett. (2016) 381:217–36. doi: 10.1016/j.canlet.2016.07.033 PMC4969225 26742462

[4] Ramakrishnan S. Sno(RNA)wing and pancreatic cancer metastasis. Gastroenterology. (2017) 153:12–4. doi: 10.1053/j.gastro.2017.05.002 28572011

[5] Hartupee C Nagalo BM Chabu CY Tesfay MZ Coleman-Barnett J West JT. Pancreatic cancer tumor microenvironment is a major therapeutic barrier and target. Front Immunol. (2024) 15:1287459/full. doi: 10.3389/fimmu.2024.1287459/full 38361931 PMC10867137

[6] Joseph AM Al Aiyan A Al-Ramadi B Singh SK Kishore U. Innate and adaptive immune-directed tumour microenvironment in pancreatic ductal adenocarcinoma. Front Immunol. (2024) 15:1323198/full. doi: 10.3389/fimmu.2024.1323198/full 38384463 PMC10879611

[7] Zhao Y Qin C Lin C Li Z Zhao B Li T. Pancreatic ductal adenocarcinoma cells reshape the immune microenvironment: Molecular mechanisms and therapeutic targets. Biochim Biophys Acta (BBA) - Rev Cancer. (2024) 1879:189183. doi: 10.1016/j.bbcan.2024.189183 39303859

[8] Storz P. Acinar cell plasticity and development of pancreatic ductal adenocarcinoma. Nat Rev Gastroenterol Hepatol. (2017) 14:296–304. doi: 10.1038/nrgastro.2017.12 28270694 PMC6036907

[9] Hong SM Heaphy CM Shi C Eo SH Cho H Meeker AK. Telomeres are shortened in acinar-to-ductal metaplasia lesions associated with pancreatic intraepithelial neoplasia but not in isolated acinar-to-ductal metaplasias. Modern Pathol. (2011) 24:256–66. doi: 10.1038/modpathol.2010.199 PMC3166222 20871595

[10] Distler M Aust D Weitz J Pilarsky C Grützmann R. Precursor lesions for sporadic pancreatic cancer: PanIN, IPMN, and MCN. BioMed Res Int. (2014) 2014:474905. doi: 10.1155/2014/474905 24783207 PMC3982269

[11] Noë M Brosens LAA. Pathology of pancreatic cancer precursor lesions. Surg Pathol Clinics. (2016) 9:561–80. doi: 10.1016/j.path.2016.07.008 27926360

[12] Saiki Y Horii A. Molecular pathology of pancreatic cancer. Pathol Int. (2014) 64:10–9. doi: 10.1111/pin.12129 24471965

[13] Sessa F Solcia E Capella C Bonato M Scarpa A Zamboni G. Intraductal papillary-mucinous tumours represent a distinct group of pancreatic neoplasms: An investigation of tumour cell differentiation and K-ras, p53, and c-erbB-2 abnormalities in 26 patients. Virchows Archiv. (1994) 425:357–67. doi: 10.1007/BF00199369 7820300

[14] Biankin AV Waddell N Kassahn KS Gingras MC Muthuswamy LB Johns AL. Pancreatic cancer genomes reveal aberrations in axon guidance pathway genes. Nature. (2012) 491:399–405. doi: 10.1038/nature11547 23103869 PMC3530898

[15] Yousef A Yousef M Chowdhury S Abdilleh K Knafl M Edelkamp P. Impact of KRAS mutations and co-mutations on clinical outcomes in pancreatic ductal adenocarcinoma. NPJ Precis Oncol. (2024) 8:1–13. doi: 10.1038/s41698-024-00441-5 38310130 PMC10838312

[16] Hruban RH van Mansfeld AD Offerhaus GJ van Weering DH Allison DC Goodman SN. K-ras oncogene activation in adenocarcinoma of the human pancreas: A study of 82 carcinomas using a combination of mutant-enriched polymerase chain reaction analysis and allele-specific oligonucleotide hybridization. Am J Pathol. (1993) 143:545–54. PMC1887038 8342602

[17] van Heek NT Meeker AK Kern SE Yeo CJ Lillemoe KD Cameron JL. Telomere shortening is nearly universal in pancreatic intraepithelial neoplasia. Am J Pathol. (2002) 161:1541–7. doi: 10.1016/S0002-9440(10)64431-5 PMC1850788 12414502

[18] Biankin AV Kench JG Morey AL Lee CS Biankin SA Head DR. Overexpression of p21(WAF1/CIP1) is an early event in the development of pancreatic intraepithelial neoplasia. Cancer Res. (2001) 61:8830–7. 11751405

[19] Jimenez RE Warshaw AL Z’graggen K Hartwig W Taylor DZ Compton CC. Sequential accumulation of K-ras mutations and p53 overexpression in the progression of pancreatic mucinous cystic neoplasms to Malignancy. Ann Surg. (1999) 230:501–509; discussion 509–511. doi: 10.1097/00000658-199910000-00007 10522720 PMC1420899

[20] Collisson EA Bailey P Chang DK Biankin AV. Molecular subtypes of pancreatic cancer. Nat Rev Gastroenterol Hepatol. (2019) 16:207–20. doi: 10.1038/s41575-019-0109-y 30718832

[21] Veenstra VL Garcia-Garijo A van Laarhoven HW Bijlsma MF. Extracellular influences: Molecular subclasses and the microenvironment in pancreatic cancer. Cancers. (2018) 10:34. doi: 10.3390/cancers10020034 29382042 PMC5836066

[22] Moffitt RA Marayati R Flate EL Volmar KE Loeza SGH Hoadley KA. Virtual microdissection identifies distinct tumor- and stroma-specific subtypes of pancreatic ductal adenocarcinoma. Nat Genet. (2015) 47:1168–78. doi: 10.1038/ng.3398 PMC4912058 26343385

[23] Klein L Tu M Krebs N Urbach L Grimm D Latif MU. Spatial tumor immune heterogeneity facilitates subtype co-existence and therapy response via AP1 dichotomy in pancreatic cancer. (2023). doi: 10.1101/2023.10.30.563552 PMC11704331 39762215

[24] Krebs N Klein L Wegwitz F Espinet E Maurer HC Tu M. Axon guidance receptor ROBO3 modulates subtype identity and prognosis via AXL-associated inflammatory network in pancreatic cancer. JCI Insight. (2022) 7:e154475. doi: 10.1172/jci.insight.154475 35993361 PMC9462476

[25] Tu M Klein L Espinet E Georgomanolis T Wegwitz F Li X. TNF-α-producing macrophages determine subtype identity and prognosis via AP1 enhancer reprogramming in pancreatic cancer. Nat Cancer. (2021) 2:1185–203. doi: 10.1038/s43018-021-00253-4 35122059

[26] Puleo F Nicolle R Blum Y Cros J Marisa L Demetter P. Stratification of pancreatic ductal adenocarcinomas based on tumor and microenvironment features. Gastroenterology. (2018) 155:1999–2013.e3. doi: 10.1053/j.gastro.2018.08.033 30165049

[27] Espinet E Klein L Puré E Singh SK. Mechanisms of PDAC subtype heterogeneity and therapy response. Trends Cancer. (2022) 8:1060–71. doi: 10.1016/j.trecan.2022.08.005 36117109

[28] Juiz N Elkaoutari A Bigonnet M Gayet O Roques J Nicolle R. Basal-like and classical cells coexist in pancreatic cancer revealed by single-cell analysis on biopsy-derived pancreatic cancer organoids from the classical subtype. FASEB J. (2020) 34:12214–28. doi: 10.1096/fj.202000829R 32686876

[29] Grünwald BT Devisme A Andrieux G Vyas F Aliar K McCloskey CW. Spatially confined sub-tumor microenvironments in pancreatic cancer. Cell. (2021) 184:5577–5592.e18. doi: 10.1016/j.cell.2021.10.019 34644529

[30] Raghavan S Winter PS Navia AW Williams HL DenAdel A Lowder KE. Microenvironment drives cell state, plasticity, and drug response in pancreatic cancer. Cell. (2021) 184:6119–37. doi: 10.1016/j.cell.2021.11.019 PMC8822455 34890551

[31] Werba G Weissinger D Kawaler EA Zhao E Kalfakakou D Dhara S. Single-cell RNA sequencing reveals the effects of chemotherapy on human pancreatic adenocarcinoma and its tumor microenvironment. Nat Commun. (2023) 14:797. doi: 10.1038/s41467-023-36494-8 36781852 PMC9925748

[32] Cancer Genome Atlas Research Network. Integrated genomic characterization of pancreatic ductal adenocarcinoma. Cancer Cell. (2017) 32:185–203.e13. doi: 10.1016/j.ccell.2017.07.007 28810144 PMC5964983

[33] Waters AM Der CJ. KRAS: The critical driver and therapeutic target for pancreatic cancer. Cold Spring Harbor Perspect Med. (2018) 8:a031435. doi: 10.1101/cshperspect.a031435 PMC5995645 29229669

[34] Iacobuzio-Donahue CA. Genetic evolution of pancreatic cancer: Lessons learnt from the pancreatic cancer genome sequencing project. Gut. (2012) 61:1085–94. doi: 10.1136/gut.2010.236026 PMC3356493 21749982

[35] Witkiewicz AK McMillan EA Balaji U Baek G Lin WC Mansour J. Whole-exome sequencing of pancreatic cancer defines genetic diversity and therapeutic targets. Nat Commun. (2015) 6:6744. doi: 10.1038/ncomms7744 25855536 PMC4403382

[36] Raphael BJ Hruban RH Aguirre AJ Moffitt RA Yeh JJ Stewart C. Integrated genomic characterization of pancreatic ductal adenocarcinoma. Cancer Cell. (2017) 32:185–203.e13. doi: 10.1016/j.ccell.2017.07.007 28810144 PMC5964983

[37] Waddell N Pajic M Patch AM Chang DK Kassahn KS Bailey P. Whole genomes redefine the mutational landscape of pancreatic cancer. Nature. (2015) 518:495–501. doi: 10.1038/nature14169 25719666 PMC4523082

[38] Moskaluk CA Hruban RH Kern SE. p16 and K-ras gene mutations in the intraductal precursors of human pancreatic adenocarcinoma. Cancer Res. (1997) 57:2140–3. 9187111

[39] Digiuseppe JA Hruban RH. Pathobiology of cancer of the pancreas. Semin Surg Oncol. (1995) 11:87–96. doi: 10.1002/ssu.2980110203

[40] Dardare J Witz A Merlin JL Gilson P Harlé A. SMAD4 and the TGFβ pathway in patients with pancreatic ductal adenocarcinoma. Int J Mol Sci. (2020) 21:3534. doi: 10.3390/ijms21103534 32429474 PMC7278913

[41] Bartsch DK Sina-Frey M Lang S Wild A Gerdes B Barth P. CDKN2A germline mutations in familial pancreatic cancer. Ann Surg. (2002) 236:730–7. doi: 10.1097/00000658-200212000-00005 PMC1422639 12454511

[42] He P Yang JW Yang VW Bialkowska AB. Krüppel-like factor 5, increased in pancreatic ductal adenocarcinoma, promotes proliferation, acinar-to-ductal metaplasia, pancreatic intraepithelial neoplasia, and tumor growth in mice. Gastroenterology. (2018) 154:1494–1508.e13. doi: 10.1053/j.gastro.2017.12.009 29248441 PMC5880723

[43] Jones S Zhang X Parsons DW Lin JCH Leary RJ Angenendt P. Core signaling pathways in human pancreatic cancers revealed by global genomic analyses. Science. (2008) 321:1801–6. doi: 10.1126/science.1164368 PMC2848990 18772397

[44] Bournet B Buscail C Muscari F Cordelier P Buscail L. Targeting KRAS for diagnosis, prognosis, and treatment of pancreatic cancer: Hopes and realities. Eur J Cancer. (2016) 54:75–83. doi: 10.1016/j.ejca.2015.11.012 26735353

[45] Haigis KM. KRAS alleles: The devil is in the detail. Trends Cancer. (2017) 3:686–97. doi: 10.1016/j.trecan.2017.08.006 PMC5824632 28958387

[46] Buscail L Bournet B Cordelier P. Role of oncogenic KRAS in the diagnosis, prognosis, and treatment of pancreatic cancer. Nat Rev Gastroenterol Hepatol. (2020) 17:153–68. doi: 10.1038/s41575-019-0245-4 32005945

[47] Aguirre AJ Bardeesy N Sinha M Lopez L Tuveson DA Horner J. Activated Kras and Ink4a/Arf deficiency cooperate to produce metastatic pancreatic ductal adenocarcinoma. Genes Dev. (2003) 17:3112–26. doi: 10.1101/gad.1158703 PMC305262 14681207

[48] Hingorani SR Petricoin EF Maitra A Rajapakse V King C Jacobetz MA. Preinvasive and invasive ductal pancreatic cancer and its early detection in the mouse. Cancer Cell. (2003) 4:437–50. doi: 10.1016/S1535-6108(03)00270-8 14706336

[49] Hingorani SR Wang L Multani AS Combs C Deramaudt TB Hruban RH. Trp53R172H and KrasG12D cooperate to promote chromosomal instability and widely metastatic pancreatic ductal adenocarcinoma in mice. Cancer Cell. (2005) 7:469–83. doi: 10.1016/j.ccr.2005.04.023 15894267

[50] Kojima K Vickers SM Adsay NV Jhala NC Kim HG Schoeb TR. Inactivation of Smad4 accelerates KrasG12D-mediated pancreatic neoplasia. Cancer Res. (2007) 67:8121–30. doi: 10.1158/0008-5472.CAN-07-1342 17804724

[51] Bardeesy N Aguirre AJ Chu GC Cheng KH Lopez LV Hezel AF. Both p16Ink4a and the p19Arf-p53 pathway constrain progression of pancreatic adenocarcinoma in the mouse. Proc Natl Acad Sci. (2006) 103:5947–52. doi: 10.1073/pnas.0601273103 PMC1458678 16585505

[52] Voutsadakis IA. Mutations of p53 associated with pancreatic cancer and therapeutic implications. Ann Hepato-Biliary-Pancreatic Surg. (2021) 25:315–27. doi: 10.14701/ahbps.2021.25.3.315 PMC8382872 34402431

[53] Kim WY Sharpless NE. The regulation of INK4/ARF in cancer and aging. Cell. (2006) 127:265–75. doi: 10.1016/j.cell.2006.10.003 17055429

[54] Bertoli C Skotheim JM de Bruin RAM. Control of cell cycle transcription during G1 and S phases. Nat Rev Mol Cell Biol. (2013) 14:518–28. doi: 10.1038/nrm3629 PMC4569015 23877564

[55] Sharpless NE DePinho RA. The INK4A/ARF locus and its two gene products. Curr Opin Genet Dev. (1999) 9:22–30. doi: 10.1016/S0959-437X(99)80004-5 10072356

[56] Yachida S White CM Naito Y Zhong Y Brosnan JA Macgregor-Das AM. Clinical significance of the genetic landscape of pancreatic cancer and implications for identification of potential long-term survivors. Clin Cancer Res. (2012) 18:633947. doi: 10.1158/1078-0432.CCR-12-1215 PMC3500447 22991414

[57] AACR Project GENIE Consortium. AACR Project GENIE: Powering precision medicine through an international consortium. Cancer Discovery. (2017) 7:818–31. doi: 10.1158/2159-8290.CD-17-0151 PMC5611790 28572459

[58] Hahn SA Schutte M Hoque AT Moskaluk CA da Costa LT Rozenblum E. DPC4, a candidate tumor suppressor gene at human chromosome 18q21.1. Science. (1996) 271:350–3. doi: 10.1126/science.271.5247.350 8553070

[59] Kubiczkova L Sedlarikova L Hajek R Sevcikova S. TGF-β—An excellent servant but a bad master. J Trans Med. (2012) 10:183. doi: 10.1186/1479-5876-10-183 PMC3494542 22943793

[60] Bailey P Chang DK Nones K Johns AL Patch AM Gingras MC. Genomic analyses identify molecular subtypes of pancreatic cancer. Nature. (2016) 531:47–52. doi: 10.1038/nature16965 26909576

[61] Pietrobono S Bertolini M De Vita V Sabbadini F Fazzini F Frusteri C. CCL3 predicts exceptional response to TGFβ inhibition in basal-like pancreatic cancer enriched in LIF-producing macrophages. NPJ Precis Oncol. (2024) 8:246. doi: 10.1038/s41698-024-00742-3 39478186 PMC11525688

[62] Ellenrieder V Buck A Harth A Jungert K Buchholz M Adler G. KLF11 mediates a critical mechanism in TGF-beta signaling that is inactivated by Erk-MAPK in pancreatic cancer cells. Gastroenterology. (2004) 127:607–20. doi: 10.1053/j.gastro.2004.05.018 15300592

[63] Roberts NJ Jiao Y Yu J Kopelovich L Petersen GM Bondy ML. ATM mutations in patients with hereditary pancreatic cancer. Cancer Discovery. (2012) 2:416. doi: 10.1158/2159-8290.CD-11-0194 PMC3676748 22585167

[64] Armstrong SA Schultz CW Azimi-Sadjadi A Brody JR Pishvaian MJ. ATM dysfunction in pancreatic adenocarcinoma and associated therapeutic implications. Mol Cancer Ther. (2019) 18:1899–908. doi: 10.1158/1535-7163.MCT-19-0208 PMC6830515 31676541

[65] Makohon-Moore AP Zhang M Reiter JG Bozic I Allen B Kundu D. Limited heterogeneity of known driver gene mutations among the metastases of individual patients with pancreatic cancer. Nat Genet. (2017) 49:358–66. doi: 10.1038/ng.3764 PMC5663439 28092682

[66] Jaworski JJ Morgan RD Sivakumar S. Circulating cell-free tumor DNA for early detection of pancreatic cancer. Cancers (Basel). (2020) 12:3704. doi: 10.3390/cancers12123704 33317202 PMC7763954

[67] Kustanovich A Schwartz R Peretz T Grinshpun A. Life and death of circulating cell-free DNA. Cancer Biol Ther. (2019) 20:1057–67. doi: 10.1080/15384047.2019.1598759 PMC6606043 30990132

[68] Tug S Helmig S Deichmann ER Schmeier-Jürchott A Wagner E Zimmermann T. Exercise-induced increases in cell-free DNA in human plasma originate predominantly from cells of the hematopoietic lineage. Exercise Immunol Rev. (2015) 21:164–73. 25826002

[69] Iovanna J. Implementing biological markers as a tool to guide clinical care of patients with pancreatic cancer. Trans Oncol. (2021) 14:100965. doi: 10.1016/j.tranon.2020.100965 PMC7704461 33248412

[70] Cheng F Su L Qian C. Circulating tumor DNA: A promising biomarker in the liquid biopsy of cancer. Oncotarget. (2016) 7:48832–41. doi: 10.18632/oncotarget.9453 PMC5217053 27223063

[71] Lo YMD Zhang J Leung TN Lau TK Chang AM Hjelm NM. Rapid clearance of fetal DNA from maternal plasma. Am J Hum Genet. (1999) 64:218–24. doi: 10.1086/302205 PMC1377720 9915961

[72] Diehl F Schmidt K Choti MA Romans K Goodman S Li M. Circulating mutant DNA to assess tumor dynamics. Nat Med. (2008) 14:985–90. doi: 10.1038/nm.1789 PMC2820391 18670422

[73] Sausen M Phallen J Adleff V Jones S Leary RJ Barrett MT. Clinical implications of genomic alterations in the tumor and circulation of pancreatic cancer patients. Nat Commun. (2015) 6:7686. doi: 10.1038/ncomms8686 26154128 PMC4634573

[74] Pietrasz D Pécuchet N Garlan F Didelot A Dubreuil O Doat S. Plasma circulating tumor DNA in pancreatic cancer patients is a prognostic marker. Clin Cancer Res. (2017) 23:116–23. doi: 10.1158/1078-0432.CCR-16-0806 27993964

[75] Jiang J Ye S Xu Y Chang L Hu X Ru G. Circulating tumor DNA as a potential marker to detect minimal residual disease and predict recurrence in pancreatic cancer. Front Oncol. (2020) 10:1220. doi: 10.3389/fonc.2020.01220 32850360 PMC7406781

[76] Toledano-Fonseca M Cano MT Inga E Rodríguez-Alonso R Gómez-España MA Guil-Luna S. Circulating cell-free DNA-based liquid biopsy markers for the non-invasive prognosis and monitoring of metastatic pancreatic cancer. Cancers. (2020) 12:1754. doi: 10.3390/cancers12071754 32630266 PMC7409337

[77] Cao F Wei A Hu X He Y Zhang J Xia L. Integrated epigenetic biomarkers in circulating cell-free DNA as a robust classifier for pancreatic cancer. Clin Epigenet. (2020) 12:112. doi: 10.1186/s13148-020-00898-2 PMC7376965 32703318

[78] Guler GD Ning Y Ku C-J Phillips T McCarthy E Ellison CK. Detection of early-stage pancreatic cancer using 5-hydroxymethylcytosine signatures in circulating cell-free DNA. Nat Commun. (2020) 11:5270. doi: 10.1038/s41467-020-18965-w 33077732 PMC7572413

[79] Khan IA Rashid S Singh N Rashid S Singh V Gunjan D. Panel of serum miRNAs as potential non-invasive biomarkers for pancreatic ductal adenocarcinoma. Sci Rep. (2021) 11:2824. doi: 10.1038/s41598-021-82053-z 33531550 PMC7854650

[80] Bukys T Kurlinkus B Sileikis A Vitkus D. The prospect of improving pancreatic cancer diagnostic capabilities by implementing blood biomarkers: A study of evaluating properties of a single IL-8 and in conjunction with CA19-9, CEA, and CEACAM6. Biomedicines. (2024) 12:2344. doi: 10.3390/biomedicines12102344 39457656 PMC11505492

[81] Gillson JE Byeon S Chou A Maloney S Pavlakis N Clarke SJ. Promising biomarker panel to monitor therapeutic efficacy of neoadjuvant chemotherapy in pancreatic cancer patients. Eur J Clin Invest. (2024). doi: 10.1111/eci.14341 39487743

[82] Vitello DJ Shah D Wells A Masnyk L Cox M Janczewski LM. Mutant KRAS in circulating tumor DNA as a biomarker in localized pancreatic cancer in patients treated with neoadjuvant chemotherapy. Ann Surg. (2024). doi: 10.1097/SLA.0000000000006562 39471087

[83] Diab M Muqbil I Mohammad RM Azmi AS Philip PA. The role of microRNAs in the diagnosis and treatment of pancreatic adenocarcinoma. J Clin Med. (2016) 5:59. doi: 10.3390/jcm5060059 27322337 PMC4929414

[84] Engels MML Berger CK Mahoney DW Hoogenboom SA Sarwal D Klatte DCF. Multimodal pancreatic cancer detection using methylated DNA biomarkers in pancreatic juice and plasma CA 19-9: A prospective multicenter study. Clin Gastroenterol Hepatol Assoc. (2024) S1542-3565(24)009741. doi: 10.1016/j.cgh.2024.09.074 PMC11930620 39477082

[85] Wang B Sun S Liu Z. Analysis of dysregulation of immune system in pancreatic cancer based on gene expression profile. Mol Biol Rep. (2014) 41:4361–7. doi: 10.1007/s11033-014-3292-7 24619357

[86] Wu J Li Z Zeng K Wu K Xu D Zhou J. Key genes associated with pancreatic cancer and their association with outcomes: A bioinformatics analysis. Mol Med Rep. (2019) 20:1343–52. doi: 10.3892/mmr.2019.10355 31173193

[87] Eyuboglu S Alpsoy S Uversky VN Coskuner-Weber O. Key genes and pathways in the molecular landscape of pancreatic ductal adenocarcinoma: A bioinformatics and machine learning study. Comput Biol Chem. (2024) 113:108268. doi: 10.1016/j.compbiolchem.2023.108268 39467488

[88] Waddington CH. The epigenotype. Int J Epidemiol. (2012) 41:10–3. doi: 10.1093/ije/dyr184 22186258

[89] Wang SS Xu J Ji KY Hwang CI. Epigenetic alterations in pancreatic cancer metastasis. Biomolecules. (2021) 11:1082. doi: 10.3390/biom11081082 34439749 PMC8394313

[90] Lomberk G Blum Y Nicolle R Nair A Gaonkar KS Marisa L. Distinct epigenetic landscapes underlie the pathobiology of pancreatic cancer subtypes. Nat Commun. (2018) 9:1978. doi: 10.1038/s41467-018-04383-6 29773832 PMC5958058

[91] Costa-Pinheiro P Montezuma D Henrique R Jerónimo C. Diagnostic and prognostic epigenetic biomarkers in cancer. Epigenomics. (2015) 7:1003–15. doi: 10.2217/epi.15.39 26479312

[92] Roberts NJ Norris AL Petersen GM Bondy ML Brand R Gallinger S. Whole genome sequencing defines the genetic heterogeneity of familial pancreatic cancer. Cancer Discovery. (2016) 6:166–75. doi: 10.1158/2159-8290.CD-15-0402 PMC4744563 26658419

[93] Dhara S Chhangawala S Chintalapudi H Askan G Aveson V Massa AL. Pancreatic cancer prognosis is predicted by an ATAC-array technology for assessing chromatin accessibility. Nat Commun. (2021) 12:3044. doi: 10.1038/s41467-021-23367-2 34031415 PMC8144607

[94] Ku B Eisenbarth D Baek S Jeong TK Kang JG Hwang D. PRMT1 promotes pancreatic cancer development and resistance to chemotherapy. Cell Rep Med. (2024) 5:101461. doi: 10.1016/j.xcrm.2024.101461 38460517 PMC10983040

[95] Zheng NN Zhou M Sun F Huai MX Zhang Y Qu CY. Combining protein arginine methyltransferase inhibitor and anti-programmed death-ligand-1 inhibits pancreatic cancer progression. World J Gastroenterol. (2020) 26:3737–49. doi: 10.3748/wjg.v26.i26.3737 PMC7383845 32774054

[96] Shao L Yu H Wang M Chen L Ji B Wu T. DKK1-SE recruits AP1 to activate the target gene DKK1 thereby promoting pancreatic cancer progression. Cell Death Dis. (2024) 15:566. doi: 10.1038/s41419-024-06648-x 39107271 PMC11303742

[97] Mortoglou M Tabin ZK Arisan ED Kocher HM Uysal-Onganer P. Non-coding RNAs in pancreatic ductal adenocarcinoma: New approaches for better diagnosis and therapy. Trans Oncol. (2021) 14:101090. doi: 10.1016/j.tranon.2021.101090 PMC8042452 33831655

[98] Anastasiadou E Jacob LS Slack FJ. Non-coding RNA networks in cancer. Nat Rev Cancer. (2018) 18:5–18. doi: 10.1038/nrc.2017.99 29170536 PMC6337726

[99] Huang X Zhi X Gao Y Ta N Jiang H Zheng J. LncRNAs in pancreatic cancer. Oncotarget. (2016) 7:57379–90. doi: 10.18632/oncotarget.10545 PMC5302996 27429196

[100] Pang E-J Yang R Fu X Liu Y. Overexpression of long non-coding RNA MALAT1 is correlated with clinical progression and unfavorable prognosis in pancreatic cancer. Tumour Biol. (2015) 36:2403–7. doi: 10.1007/s13277-014-2850-8 25481511

[101] Tang Q Hann SS. HOTAIR: An oncogenic long non-coding RNA in human cancer. Cell Physiol Biochem. (2018) 47:893–913. doi: 10.1159/000490131 29843138

[102] Maekawa T Fukaya R Takamatsu S Itoyama S Fukuoka T Yamada M. Possible involvement of Enterococcus infection in the pathogenesis of chronic pancreatitis and cancer. Biochem Biophys Res Commun. (2018) 506:962–9. doi: 10.1016/j.bbrc.2018.10.169 30401562

[103] Li P Zhang H Dai M. Current status and prospect of gut and oral microbiome in pancreatic cancer: Clinical and translational perspectives. Cancer Lett. (2024) 604:217274. doi: 10.1016/j.canlet.2024.217274 39307411

[104] Pushalkar S Hundeyin M Daley D Zambirinis CP Kurz E Mishra A. The pancreatic cancer microbiome promotes oncogenesis by induction of innate and adaptive immune suppression. Cancer Discovery. (2018) 8:403–16. doi: 10.1158/2159-8290.CD-17-1134 PMC6225783 29567829

[105] Wolpin BM Bao Y Qian ZR Wu C Kraft P Ogino S. Hyperglycemia, insulin resistance, impaired pancreatic β-cell function, and risk of pancreatic cancer. JNCI: J Natl Cancer Institute. (2013) 105:1027–35. doi: 10.1093/jnci/djt123 PMC3714020 23847240

[106] Andersen DK Korc M Petersen GM Eibl G Li D Rickels MR. Diabetes, pancreatogenic diabetes, and pancreatic cancer. Diabetes. (2017) 66:1103–10. doi: 10.2337/db16-1477 PMC5399609 28507210

[107] Pergamo M Miller G. Myeloid-derived suppressor cells and their role in pancreatic cancer. Cancer Gene Ther. (2017) 24:100–5. doi: 10.1038/cgt.2016.47 27910857

[108] Weber R Groth C Lasser S Arkhypov I Petrova V Altevogt P. IL-6 as a major regulator of MDSC activity and possible target for cancer immunotherapy. Cell Immunol. (2021) 359:104254. doi: 10.1016/j.cellimm.2020.104254 33296753

[109] Zhang Y Velez-Delgado A Mathew E Li D Mendez FM Flannagan K. Myeloid cells are required for PD-1/PD-L1 checkpoint activation and the establishment of an immunosuppressive environment in pancreatic cancer. Gut. (2016) 66:124. doi: 10.1136/gutjnl-2016-312078 27402485 PMC5256390

[110] Bronte V Brandau S Chen SH Colombo MP Frey AB Greten TF. Recommendations for myeloid-derived suppressor cell nomenclature and characterization standards. Nat Commun. (2016) 7:12150. doi: 10.1038/ncomms12150 27381735 PMC4935811

[111] Porembka MR Mitchem JB Belt BA Hsieh CS Lee HM Herndon J. Pancreatic adenocarcinoma induces bone marrow mobilization of myeloid-derived suppressor cells which promote primary tumor growth. Cancer Immunol Immunother. (2012) 61:1373–85. doi: 10.1007/s00262-012-1198-0 PMC3697836 22215137

[112] Bayne LJ Beatty GL Jhala N Clark CE Rhim AD Stanger BZ. Tumor-derived granulocyte-macrophage colony-stimulating factor regulates myeloid inflammation and T cell immunity in pancreatic cancer. Cancer Cell. (2012) 21:822–35. doi: 10.1016/j.ccr.2012.04.025 PMC3575028 22698406

[113] Corzo CA Condamine T Lu L Cotter MJ Youn JI Cheng P. HIF-1α regulates function and differentiation of myeloid-derived suppressor cells in the tumor microenvironment. J Exp Med. (2010) 207:2439–53. doi: 10.1084/jem.20100587 PMC2964584 20876310

[114] Kumar V Patel S Tcyganov E Gabrilovich DI. The nature of myeloid-derived suppressor cells in the tumor microenvironment. Trends Immunol. (2016) 37:208–20. doi: 10.1016/j.it.2016.01.004 PMC4775398 26858199

[115] Hemida AS Ahmed MM Tantawy MS. HOXA9 and CD163 potentiate pancreatic ductal adenocarcinoma progression. Diagn Pathol. (2024) 19:141. doi: 10.1186/s13000-024-01303-6 39462379 PMC11514874

[116] Mantovani A Marchesi F Malesci A Laghi L Allavena P. Tumour-associated macrophages as treatment targets in oncology. Nat Rev Clin Oncol. (2017) 14:399–416. doi: 10.1038/nrclinonc.2017.41 28117416 PMC5480600

[117] Li Q Liu Y Zhi R Wang Y. The prognostic significance of twist in pancreatic cancer and its role in cancer promotion through the regulation of the immune microenvironment and EMT mechanisms. Discovery Oncol. (2024) 15:593. doi: 10.1186/s13046-024-00398-3 PMC11512955 39460846

[118] Marigo I Dolcetti L Serafini P Zanovello P Bronte V. Tumor-induced tolerance and immune suppression by myeloid-derived suppressor cells. Immunol Rev. (2008) 222:162–79. doi: 10.1111/j.1600-065X.2008.00616.x 18364001

[119] Batlle E Massagué J. Transforming growth factor-β signaling in immunity and cancer. Immunity. (2019) 50:924–40. doi: 10.1016/j.immuni.2019.03.024 PMC7507121 30995507

[120] Chen ML Pittet MJ Gorelik L Flavell RA Weissleder R von Boehmer H. Regulatory T cells suppress tumor-specific CD8 T cell cytotoxicity through TGF-β signals *in vivo* . Proc Natl Acad Sci U.S.A. (2005) 102:419–24. doi: 10.1073/pnas.0408885102 PMC544311 15623559

[121] Hiraoka N Onozato K Kosuge T Hirohashi S. Prevalence of FOXP3+ regulatory T cells increases during the progression of pancreatic ductal adenocarcinoma and its premalignant lesions. Clin Cancer Res. (2006) 12:5423–34. doi: 10.1158/1078-0432.CCR-06-1101 17000676

[122] Wartenberg M Zlobec I Perren A Koelzer VH Gloor B Lugli A. Accumulation of FOXP3+ T-cells in the tumor microenvironment is associated with an epithelial-mesenchymal-transition-type tumor budding phenotype and is an independent prognostic factor in surgically resected pancreatic ductal adenocarcinoma. Oncotarget. (2015) 6:4190–201. doi: 10.18632/oncotarget.3187 PMC4414182 25669968

[123] Chen Q Yin H Liu S Shoucair S Ding N Ji Y. Prognostic value of tumor-associated N1/N2 neutrophil plasticity in patients following radical resection of pancreatic ductal adenocarcinoma. J Immunother Cancer. (2022) 10:e005798. doi: 10.1136/jitc-2022-005798 36600557 PMC9730407

[124] Jablonska J Leschner S Westphal K Lienenklaus S Weiss S. Neutrophils responsive to endogenous IFN-β regulate tumor angiogenesis and growth in a mouse tumor model. J Clin Invest. (2010) 120:1151–64. doi: 10.1172/JCI40919 PMC2846036 20237412

[125] Luo H Ikenaga N Nakata K Higashijima N Zhong P Kubo A. Tumor-associated neutrophils upregulate Nectin2 expression, creating the immunosuppressive microenvironment in pancreatic ductal adenocarcinoma. J Exp Clin Cancer Res. (2024) 43:258. doi: 10.1186/s13046-024-02529-w 39261943 PMC11389261

[126] Mishalian I Bayuh R Eruslanov E Michaeli J Levy L Zolotarov L. Neutrophils recruit regulatory T-cells into tumors via secretion of CCL17—a new mechanism of impaired antitumor immunity. Int J Cancer. (2014) 135:1178–86. doi: 10.1002/ijc.28818 24501019

[127] Fridlender ZG Sun J Kim S Kapoor V Cheng G Ling L. Polarization of tumor-associated neutrophil phenotype by TGF-beta: “N1” versus “N2” TAN. Cancer Cell. (2009) 16:183–94. doi: 10.1016/j.ccr.2009.06.017 PMC2754404 19732719

[128] Bachem MG Schünemann M Ramadani M Siech M Beger H Buck A. Pancreatic carcinoma cells induce fibrosis by stimulating proliferation and matrix synthesis of stellate cells. Gastroenterology. (2005) 128:907–21. doi: 10.1053/j.gastro.2005.01.018 15825074

[129] Manoukian P Bijlsma M van Laarhoven H. The cellular origins of cancer-associated fibroblasts and their opposing contributions to pancreatic cancer growth. Front Cell Dev Biol. (2021) 9:743907. doi: 10.3389/fcell.2021.743907 34646829 PMC8502878

[130] Zhang T Ren Y Yang P Wang J Zhou H. Cancer-associated fibroblasts in pancreatic ductal adenocarcinoma. Cell Death Dis. (2022) 13:1–11. doi: 10.1038/s41419-022-04997-4 PMC9596464 36284087

[131] Sherman MH di Magliano MP. Cancer-associated fibroblasts: Lessons from pancreatic cancer. Annu Rev Cancer Biol. (2023) 7:43–55. doi: 10.1146/annurev-cancerbio-051222-125845

[132] Sun Y Qiao Y Niu Y Madhavan BK Fang C Hu J. ARP2/3 complex affects myofibroblast differentiation and migration in pancreatic ductal adenocarcinoma. Int J Cancer. (2024) 156:12721281. doi: 10.1002/ijc.37021 PMC11737003 39472297

[133] Wehr AY Furth EE Sangar V Blair IA Yu KH. Analysis of the human pancreatic stellate cell secreted proteome. Pancreas. (2011) 40:557–66. doi: 10.1097/MPA.0b013e318207f376 PMC3086313 21499210

[134] Jacobetz MA Chan DS Neesse A Bapiro TE Cook N Frese KK. Hyaluronan impairs vascular function and drug delivery in a mouse model of pancreatic cancer. Gut. (2013) 62:112–20. doi: 10.1136/gutjnl-2011-301129 PMC3551211 22466618

[135] Provenzano PP Cuevas C Chang AE Goel VK Von Hoff DD Hingorani SR. Enzymatic targeting of the stroma ablates physical barriers to treatment of pancreatic ductal adenocarcinoma. Cancer Cell. (2012) 21:418–29. doi: 10.1016/j.ccr.2012.01.007 PMC3371414 22439937

[136] Hingorani SR Harris WP Beck JT Berdov BA Wagner SA Pshevlotsky EM. Phase Ib study of PEGylated recombinant human hyaluronidase and gemcitabine in patients with advanced pancreatic cancer. Clin Cancer Res. (2016) 22:2848–54. doi: 10.1158/1078-0432.CCR-15-1829 PMC7787348 26813359

[137] Bailey JM Swanson BJ Hamada T Eggers JP Singh PK Caffery T. Sonic hedgehog promotes desmoplasia in pancreatic cancer. Clin Cancer Res. (2008) 14:5995–6004. doi: 10.1158/1078-0432.CCR-08-0937 18829478 PMC2782957

[138] Geng X Chen H Zhao L Hu J Yang W Li G. Cancer-associated fibroblast (CAF) heterogeneity and targeting therapy of CAFs in pancreatic cancer. Front Cell Dev Biol. (2021) 9:655152. doi: 10.3389/fcell.2021.655152 34336821 PMC8319605

[139] Rhim AD Oberstein PE Thomas DH Mirek ET Palermo CF Sastra SA. Stromal elements act to restrain, rather than support, pancreatic ductal adenocarcinoma. Cancer Cell. (2014) 25:735–47. doi: 10.1016/j.ccr.2014.04.005 PMC4096698 24856585

[140] Chang DZ Ma Y Ji B Wang H Deng D Liu Y. Mast cells in the tumor microenvironment promote the *in vivo* growth of pancreatic ductal adenocarcinoma. Clin Cancer Res. (2011) 17:7015. doi: 10.1158/1078-0432.CCR-11-0732 21976550 PMC4089502

[141] Ma Y Hwang RF Logsdon CD Ullrich SE. Dynamic mast cell–stromal cell interactions promote growth of pancreatic cancer. Cancer Res. (2013) 73:3927–37. doi: 10.1158/0008-5472.CAN-13-0531 PMC3702652 23633481

[142] Gong R Ren H. Targeting chemokines/chemokine receptors: A promising strategy for enhancing the immunotherapy of pancreatic ductal adenocarcinoma. Signal Transduct Targeted Ther. (2020) 5:1–2. doi: 10.1038/s41392-020-0157-5 PMC7419510 32782241

[143] Homey B Müller A Zlotnik A. Chemokines: Agents for the immunotherapy of cancer? Nat Rev Immunol. (2002) 2:175–84. doi: 10.1038/nri746 11913068

[144] Zhang J Patel L Pienta KJ. CC chemokine ligand 2 (CCL2) promotes prostate cancer tumorigenesis and metastasis. Cytokine Growth Factor Rev. (2010) 21:41–8. doi: 10.1016/j.cytogfr.2009.12.005 PMC2857769 20005149

[145] Kishore U Varghese PM Mangogna A Klein L Tu M Urbach L. Neutrophil-derived complement factor P induces cytotoxicity in basal-like cells via caspase 3/7 activation in pancreatic cancer. bioRxiv. (2023) 10.28.564512. doi: 10.1101/2023.10.28.564512v1

[146] Kaur A Riaz MS Singh SK Kishore U. Human surfactant protein D suppresses epithelial-to-mesenchymal transition in pancreatic cancer cells by downregulating TGF-β. Front Immunol. (2018) 9:1844. doi: 10.3389/fimmu.2018.01844 30158928 PMC6104167

[147] Kaur A Riaz MS Murugaiah V Varghese PM Singh SK Kishore U. A recombinant fragment of human surfactant protein D induces apoptosis in pancreatic cancer cell lines via Fas-mediated pathway. Front Immunol. (2018) 9:1126. doi: 10.3389/fimmu.2018.01126 29915574 PMC5994421

[148] Yang J Lin P Yang M Liu W Fu X Liu D. Integrated genomic and transcriptomic analysis reveals unique characteristics of hepatic metastases and pro-metastatic role of complement C1q in pancreatic ductal adenocarcinoma. Genome Biol. (2021) 22:4. doi: 10.1186/s13059-020-02222-w 33397441 PMC7780398

[149] Khaliq AM Rajamohan M Saeed O Mansouri K Adil A Zhang C. Spatial transcriptomic analysis of primary and metastatic pancreatic cancers highlights tumor microenvironmental heterogeneity. Nat Genet. (2024) 56:2455–65. doi: 10.1038/s41588-024-01914-4 39294496

[150] Downward J. Targeting RAS signalling pathways in cancer therapy. Nat Rev Cancer. (2003) 3:11–22. doi: 10.1038/nrc991 12509763

[151] Melisi D Xia Q Paradiso G Ling J Moccia T Carbone C. Modulation of pancreatic cancer chemoresistance by inhibition of TAK1. J Natl Cancer Institute. (2011) 103:1190–204. doi: 10.1093/jnci/djr243 PMC3149044 21743023

[152] Huang X Li M Hou S Tian B. Role of the microbiome in systemic therapy for pancreatic ductal adenocarcinoma (Review). Int J Oncol. (2021) 59:101. doi: 10.3892/ijo.2021.5361 34738624 PMC8577795

[153] Nejman D Livyatan I Fuks G Gavert N Zwang Y Geller LT. The human tumor microbiome is composed of tumor type-specific intracellular bacteria. Science. (2020) 368:973–80. doi: 10.1126/science.aaw0125 PMC7757858 32467386

[154] Vande Voorde J Sabuncuoğlu S Noppen S Hofer A Ranjbarian F Fieuws S. Nucleoside-catabolizing enzymes in mycoplasma-infected tumor cell cultures compromise the cytostatic activity of the anticancer drug gemcitabine. J Biol Chem. (2014) 289:13054–65. doi: 10.1074/jbc.M114.552098 PMC4036319 24668817

[155] Guo W Zhang Y Guo S Mei Z Liao H Dong H. Tumor microbiome contributes to an aggressive phenotype in the basal-like subtype of pancreatic cancer. Commun Biol. (2021) 4:1019. doi: 10.1038/s42003-021-02296-3 34465850 PMC8408135

[156] Kesh K Mendez R Abdelrahman L Banerjee S Banerjee S. Type 2 diabetes induced microbiome dysbiosis is associated with therapy resistance in pancreatic adenocarcinoma. Microbial Cell Factories. (2020) 19:75. doi: 10.1186/s12934-020-01411-2 32204699 PMC7092523

[157] Kamphorst JJ Nofal M Commisso C Hackett SR Lu W Grabocka E. Human pancreatic cancer tumors are nutrient poor and tumor cells actively scavenge extracellular protein. Cancer Res. (2015) 75:544–53. doi: 10.1158/0008-5472.CAN-14-1426 PMC4316379 25644265

[158] Corbet C Feron O. Tumour acidosis: From the passenger to the driver’s seat. Nat Rev Cancer. (2017) 17:577–93. doi: 10.1038/nrc.2017.69 28912578

[159] Evan T Wang VMY Behrens A. The roles of intratumour heterogeneity in the biology and treatment of pancreatic ductal adenocarcinoma. Oncogene. (2022) 41:4686–95. doi: 10.1038/s41388-022-02238-1 PMC9568427 36088504

[160] Hermann PC Huber SL Herrler T Aicher A Ellwart JW Guba M. Distinct populations of cancer stem cells determine tumor growth and metastatic activity in human pancreatic cancer. Cell Stem Cell. (2007) 1:313–23. doi: 10.1016/j.stem.2007.06.002 18371365

[161] Li C Heidt DG Dalerba P Burant CF Zhang L Adsay V. Identification of pancreatic cancer stem cells. Cancer Res. (2007) 67:1030–7. doi: 10.1158/0008-5472.CAN-06-2210 17283135

[162] Wang VMY Ferreira RMM Almagro J Evan T Legrave N Zaw Thin M. CD9 identifies pancreatic cancer stem cells and modulates glutamine metabolism to fuel tumour growth. Nat Cell Biol. (2019) 21:1425–35. doi: 10.1038/s41556-019-0362-1 PMC6944508 31685994

[163] Fox RG Lytle NK Jaquish DV Park FD Ito T Bajaj J. Image-based detection and targeting of therapy resistance in pancreatic adenocarcinoma. Nature. (2016) 534:407–11. doi: 10.1038/nature17961 PMC4998062 27281208

[164] Bubin R Uljanovs R Strumfa I. Cancer stem cells in pancreatic ductal adenocarcinoma. Int J Mol Sci. (2023) 24:7030. doi: 10.3390/ijms24087030 37108193 PMC10138709

[165] Klein L Tu M Krebs N Urbach L Grimm D Latif MU. Spatial tumor immune heterogeneity facilitates subtype co-existence and therapy response in pancreatic cancer. Nat Commun. (2025) 16:335. doi: 10.1038/s41467-024-39123-4 39762215 PMC11704331

[166] Ligorio M Sil S Malagon-Lopez J Nieman LT Misale S Pilato MD. Stromal microenvironment shapes the intratumoral architecture of pancreatic cancer. Cell. (2019) 178:160–75. doi: 10.1016/j.cell.2019.05.023 PMC6697165 31155233

[167] Oh K Yoo YJ Torre-Healy LA Rao M Fassler D Wang P. Coordinated single-cell tumor microenvironment dynamics reinforce pancreatic cancer subtype. Nat Commun. (2023) 14:5226. doi: 10.1038/s41467-023-41004-2 37633924 PMC10460409

[168] Beatty GL Werba G Lyssiotis CA Simeone DM. The biological underpinnings of therapeutic resistance in pancreatic cancer. Genes Dev. (2021) 35:940–62. doi: 10.1101/gad.346120.121 PMC8247606 34117095

[169] Whipple AO. Pancreaticoduodenectomy for Islet carcinoma: A five-year follow-up. Ann Surg. (1945) 121:847–52. doi: 10.1097/00000658-194506000-00002 PMC1618156 17858621

[170] Jiang X Yu Z Ma Z Deng H Ren W Shi W. Superior mesenteric artery first approach can improve the clinical outcomes of pancreaticoduodenectomy: A meta-analysis. Int J Surg. (2020) 73:14–24. doi: 10.1016/j.ijsu.2019.11.019 31751791

[171] Pędziwiatr M Pisarska M Małczak P Major P Wierdak D Radkowiak D. Laparoscopic uncinate process first pancreatoduodenectomy-feasibility study of a modified “artery first” approach to pancreatic head cancer. Langenbeck’s Arch Surg. (2017) 402:917–23. doi: 10.1007/s00423-017-1594-z PMC5563330 28699023

[172] Rosso E Zimmitti G Iannelli A Garatti M. The “TRIANGLE operation” by laparoscopy: Radical pancreaticoduodenectomy with major vascular resection for borderline resectable pancreatic head cancer. Ann Surg Oncol. (2020) 27:1613–4. doi: 10.1245/s10434-019-07817-6 31802299

[173] Hackert T Strobel O Michalski CW Mihaljevic AL Mehrabi A Müller-Stich B. The TRIANGLE operation - radical surgery after neoadjuvant treatment for advanced pancreatic cancer: A single arm observational study. HPB. (2017) 19:1001–7. doi: 10.1016/j.hpb.2017.07.004 28838632

[174] Terasaki F Fukami Y Maeda A Takayama Y Takahashi T Uji M. Comparison of end-to-end anastomosis and interposition graft during pancreatoduodenectomy with portal vein reconstruction for pancreatic ductal adenocarcinoma. Langenbeck’s Arch Surg. (2019) 404:191–201. doi: 10.1007/s00423-019-01772-0 30631907

[175] Schmidt T Strobel O Schneider M Diener MK Berchtold C Mihaljevic AL. Cavernous transformation of the portal vein in pancreatic cancer surgery-venous bypass graft first. Langenbeck’s Arch Surg. (2020) 405:1045–50. doi: 10.1007/s00423-020-01960-7 PMC7541372 32915294

[176] Kinny-Köster B van Oosten F Habib JR Javed AA Cameron JL Lafaro KJ. Mesoportal bypass, interposition graft, and mesocaval shunt: Surgical strategies to overcome superior mesenteric vein involvement in pancreatic cancer. Surgery. (2020) 168:1048–55. doi: 10.1016/j.surg.2020.08.015 32951905

[177] Cai B Lu Z Neoptolemos JP Diener MK Li M Yin L. Sub-adventitial divestment technique for resecting artery-involved pancreatic cancer: A retrospective cohort study. Langenbeck’s Arch Surg. (2021) 406:691–701. doi: 10.1007/s00423-021-02411-1 33507403

[178] Schneider M Strobel O Hackert T Büchler MW. Pancreatic resection for cancer—the Heidelberg technique. Langenbeck’s Arch Surg. (2019) 404:1017–22. doi: 10.1007/s00423-019-01831-x 31728630

[179] Fortner JG. Regional resection of cancer of the pancreas: A new surgical approach. Surgery. (1973) 73:307–20. doi: 10.1016/S0039-6060(73)80188-7 4265314

[180] Bhayani NH Enomoto LM James BC Ortenzi G Kaifi JT Kimchi ET. Multivisceral and extended resections during pancreatoduodenectomy increase morbidity and mortality. Surgery. (2014) 155:567–74. doi: 10.1016/j.surg.2013.12.015 24524390

[181] Kulemann B Hoeppner J Wittel U Glatz T Keck T Wellner UF. Perioperative and long-term outcome after standard pancreaticoduodenectomy, additional portal vein and multivisceral resection for pancreatic head cancer. J Gastrointestinal Surg. (2015) 19:438–44. doi: 10.1007/s11605-014-2725-8 25567663

[182] Asbun HJ Moekotte AL Vissers FL Kunzler F Cipriani F Alseidi A. The Miami international evidence-based guidelines on minimally invasive pancreas resection. Ann Surg. (2020) 271:1–14. doi: 10.1097/SLA.0000000000003590 31567509

[183] Melvin WS Needleman BJ Krause KR Ellison EC. Robotic resection of pancreatic neuroendocrine tumor. J Laparoendoscopic Advanced Surg Techniques. (2003) 13:33–6. doi: 10.1089/109264203321235449 12676019

[184] Salky BA Edye M. Laparoscopic pancreatectomy. Surg Clinics North America. (1996) 76:539–45. doi: 10.1016/S0039-6109(05)70460-6 8669013

[185] Korrel M Vissers FL van Hilst J de Rooij T Dijkgraaf MG Festen S. Minimally invasive versus open distal pancreatectomy: An individual patient data meta-analysis of two randomized controlled trials. HPB. (2021) 23:323–30. doi: 10.1016/j.hpb.2020.10.022 33250330

[186] van Hilst J de Rooij T Klompmaker S Rawashdeh M Aleotti F Al-Sarireh B. Minimally invasive versus open distal pancreatectomy for ductal adenocarcinoma (DIPLOMA): A pan-European propensity score matched study. Ann Surg. (2019) 269:10–7. doi: 10.1097/SLA.0000000000002561 29099399

[187] Nickel F Haney CM Kowalewski KF Probst P Limen EF Kalkum E. Laparoscopic versus open pancreaticoduodenectomy: A systematic review and meta-analysis of randomized controlled trials. Ann Surg. (2020) 271:54–66. doi: 10.1097/SLA.0000000000003309 30973388

[188] van Hilst J de Rooij T Bosscha K Brinkman DJ van Dieren S Dijkgraaf MG. Laparoscopic versus open pancreatoduodenectomy for pancreatic or periampullary tumours (LEOPARD-2): A multicentre, patient-blinded, randomised controlled phase 2/3 trial. Lancet Gastroenterol Hepatol. (2019) 4:199–207. doi: 10.1016/S2468-1253(19)30003-8 30685489

[189] Wei K Hackert T. Surgical treatment of pancreatic ductal adenocarcinoma. Cancers. (2021) 13:1971. doi: 10.3390/cancers13081971 33923884 PMC8074119

[190] Von Hoff DD Ervin T Arena FP Chiorean EG Infante J Moore M. Increased survival in pancreatic cancer with nab-paclitaxel plus gemcitabine. New Engl J Med. (2013) 369:1691–703. doi: 10.1056/NEJMoa1304369 PMC4631139 24131140

[191] Frei E. Clinical cancer research: An embattled species. Cancer. (1982) 50:1979–92. doi: 10.1002/1097-0142(19821115)50:10<1979::AID-CNCR2820501002>3.0.CO;2-D 7127245

[192] Cunningham D Chau I Stocken DD Valle JW Smith D Steward W. Phase III randomized comparison of gemcitabine versus gemcitabine plus capecitabine in patients with advanced pancreatic cancer. J Clin Oncol. (2009) 27:5513–8. doi: 10.1200/JCO.2009.24.2446 19858379

[193] Conroy T Desseigne F Ychou M Bouché O Guimbaud R Bécouarn Y. FOLFIRINOX versus gemcitabine for metastatic pancreatic cancer. New Engl J Med. (2011) 364:1817–25. doi: 10.1056/NEJMoa1011923 21561347

[194] Carrato A Vieitez JM Benavides M Rodriguez-Garrote M Castillo A Ogalla GD. Phase I/II trial of sequential treatment of nab-paclitaxel in combination with gemcitabine followed by modified FOLFOX chemotherapy in patients with untreated metastatic exocrine pancreatic cancer: Phase I results. Eur J Cancer. (2020) 139:51–8. doi: 10.1016/j.ejca.2020.07.035 32977220

[195] Raphael MJ Raskin W Habbous S Tai X Beca J Dai WF. The association of drug-funding reimbursement with survival outcomes and use of new systemic therapies among patients with advanced pancreatic cancer. JAMA Netw Open. (2021) 4:e2133388. doi: 10.1001/jamanetworkopen.2021.33388 34779846 PMC8593760

[196] Breakstone R Almhanna K Raufi A Beard RE Leonard KL Renaud J. The Brown University Oncology Group experience with FOLFOX + nab-paclitaxel [FOLFOX-A] for metastatic and locally advanced pancreatic cancer: BrUOG-292 and BrUOG-318. Am J Clin Oncol. (2022) 45:327–32. doi: 10.1097/COC.0000000000000928 PMC9311474 35749747

[197] Wainberg ZA Melisi D Macarulla T Cid RP Chandana SR Fouchardière CDL. NALIRIFOX versus nab-paclitaxel and gemcitabine in treatment-naive patients with metastatic pancreatic ductal adenocarcinoma (NAPOLI 3): A randomised, open-label, phase 3 trial. Lancet. (2023) 402:1272–81. doi: 10.1016/S0140-6736(23)01301-4 PMC11664154 37708904

[198] Miller PN Romero-Hernandez F Calthorpe L Wang JJ Kim SS Corvera CU. Long-duration neoadjuvant therapy with FOLFIRINOX yields favorable outcomes for patients who undergo surgery for pancreatic cancer. Ann Surg Oncol. (2024) 31:6147–56. doi: 10.1245/s10434-024-15579-0 PMC11300478 38879670

[199] Diop-Frimpong B Chauhan VP Krane S Boucher Y Jain RK. Losartan inhibits collagen I synthesis and improves the distribution and efficacy of nanotherapeutics in tumors. Proc Natl Acad Sci. (2011) 108:2909–14. doi: 10.1073/pnas.1018892108 PMC3041115 21282607

[200] Murphy JE Wo JY Ryan DP Clark JW Jiang W Yeap BY. Total neoadjuvant therapy with FOLFIRINOX in combination with losartan followed by chemoradiotherapy for locally advanced pancreatic cancer: A phase 2 clinical trial. JAMA Oncol. (2019) 5:1020–7. doi: 10.1001/jamaoncol.2019.0892 PMC6547247 31145418

[201] Neoptolemos JP Palmer DH Ghaneh P Psarelli EE Valle JW Halloran CM. Comparison of adjuvant gemcitabine and capecitabine with gemcitabine monotherapy in patients with resected pancreatic cancer (ESPAC-4): A multicentre, open-label, randomised, phase 3 trial. Lancet. (2017) 389:1011–24. doi: 10.1016/S0140-6736(16)32409-1 28129987

[202] Conroy T Hammel P Hebbar M Ben Abdelghani M Wei AC Raoul J-L. FOLFIRINOX or gemcitabine as adjuvant therapy for pancreatic cancer. New Engl J Med. (2018) 379:2395–406. doi: 10.1056/NEJMoa1809775 30575490

[203] Reni M Riess H O’Reilly EM Park JO Hatoum H Saez BL. Phase III APACT trial of adjuvant nab-paclitaxel plus gemcitabine (nab-P + Gem) versus gemcitabine (Gem) alone for patients with resected pancreatic cancer (PC): Outcomes by geographic region. J Clin Oncol. (2020) 38:4515. doi: 10.1200/JCO.2020.38.15_suppl.4515

[204] van Dam JL Verkolf EMM Dekker EN Bonsing BA Bratlie SO Brosens LAA. Dutch Pancreatic Cancer Group. Perioperative or adjuvant mFOLFIRINOX for resectable pancreatic cancer (PREOPANC-3): study protocol for a multicenter randomized controlled trial. BMC Cancer. (2024) 23(1):728. doi: 10.1186/s12885-023-11141-5 PMC10405377 37550634

[205] Uesaka K Boku N Fukutomi A Okamura Y Konishi M Matsumoto I. Adjuvant chemotherapy of S-1 versus gemcitabine for resected pancreatic cancer: A phase 3, open-label, randomised, non-inferiority trial (JASPAC 01). Lancet. (2016) 388:248–57. doi: 10.1016/S0140-6736(16)30583-9 27265347

[206] Sinn M Bahra M Liersch T Gellert K Messmann H Bechstein W. CONKO-005: Adjuvant chemotherapy with gemcitabine plus erlotinib versus gemcitabine alone in patients after R0 resection of pancreatic cancer: A multicenter randomized phase III trial. J Clin Oncol. (2017) 35:3330–7. doi: 10.1200/JCO.2017.72.6463 28817370

[207] Kalser MH Ellenberg SS. Pancreatic cancer: Adjuvant combined radiation and chemotherapy following curative resection. Arch Surg. (1985) 120:899–903. doi: 10.1001/archsurg.1985.01390320023003 4015380

[208] Van Laethem JL Hammel P Mornex F Azria D Van Tienhoven G Vergauwe P. Adjuvant gemcitabine alone versus gemcitabine-based chemoradiotherapy after curative resection for pancreatic cancer: A randomized EORTC-40013-22012/FFCD-9203/GERCOR phase II study. J Clin Oncol. (2010) 28:4450–6. doi: 10.1200/JCO.2010.30.3446 PMC2988636 20837948

[209] Smeenk HG van Eijck CHJ Hop WC Erdmann J Tran KCK Debois M. Long-term survival and metastatic pattern of pancreatic and periampullary cancer after adjuvant chemoradiation or observation: Long-term results of EORTC trial 40891. Ann Surg. (2007) 246:734–40. doi: 10.1097/SLA.0b013e318156eef3 17968163

[210] Wang Y Xu Y Zheng Y Bao Y Wang P. Postoperative hepatic arterial infusion chemotherapy improved survival of pancreatic cancer after radical pancreatectomy: A retrospective study. OncoTargets Ther. (2018) 11:903–7. doi: 10.2147/OTT.S153886 PMC5826292 29503565

[211] Palta M Godfrey D Goodman KA Hoffe S Dawson LA Dessert D. Radiation therapy for pancreatic cancer: Executive summary of an ASTRO clinical practice guideline. Pract Radiat Oncol. (2019) 9:322–32. doi: 10.1016/j.prro.2019.06.016 31474330

[212] Bagley AF Ludmir EB Maitra A Minsky BD Smith GL Das P. NBTXR3, a first-in-class radioenhancer for pancreatic ductal adenocarcinoma: Report of first patient experience. Clin Trans Radiat Oncol. (2022) 33:66–9. doi: 10.1016/j.ctro.2021.12.012 PMC8783106 35097226

[213] Aguilera T Parikh P Ghaly M Hoffe S Herman J Caster J. Greco-2: A randomized, phase 2 study of stereotactic body radiation therapy (SBRT) in combination with rucosopasem (GC4711) in the treatment of locally advanced or borderline resectable nonmetastatic pancreatic cancer. J Clin Oncol. (2023) 41:TPS766. doi: 10.1200/JCO.2023.41.4_suppl.TPS766

[214] Hu ZI O’Reilly EM. Therapeutic developments in pancreatic cancer. Nat Rev Gastroenterol Hepatol. (2024) 21:7–24. doi: 10.1038/s41575-023-00840-w 37798442

[215] Zou W Wolchok JD Chen L. PD-L1 (B7-H1) and PD-1 pathway blockade for cancer therapy: Mechanisms, response biomarkers, and combinations. Sci Trans Med. (2016) 8:328rv4. doi: 10.1126/scitranslmed.aad7118 PMC4859220 26936508

[216] O’Reilly EM Oh DY Dhani N Renouf DJ Lee MA Sun W. Durvalumab with or without tremelimumab for patients with metastatic pancreatic ductal adenocarcinoma: A phase 2 randomized clinical trial. JAMA Oncol. (2019) 5:1431–8. doi: 10.1001/jamaoncol.2019.1588 PMC6647002 31318392

[217] Geboers B Timmer FEF Ruarus AH Pouw JEE Schouten E. AC Bakker J. Irreversible Electroporation and Nivolumab Combined with Intratumoral Administration of a Toll-Like Receptor Ligand, as a Means of In Vivo Vaccination for Metastatic Pancreatic Ductal Adenocarcinoma (PANFIRE-III). A Phase-I Study Protocol. Cancers (2021) 13(15):3902. doi: 10.3390/cancers13153902 34359801 PMC8345515

[218] Hughson AL Hannon G Salama NA Vrooman TG Stockwell CA Mills BN. Integrating IL-12 mRNA nanotechnology with SBRT eliminates T cell exhaustion in preclinical models of pancreatic cancer. Mol Ther – Nucleic Acids. (2024) 35:102350. doi: 10.1016/j.omtn.2024.102350 39469666 PMC11513558

[219] Wang X Li D Zhu B Hua Z. Single-cell transcriptome analysis identifies a novel tumor-associated macrophage subtype predicting better prognosis in pancreatic ductal adenocarcinoma. Front Cell Dev Biol. (2024) 12:1466767. doi: 10.3389/fcell.2024.1466767 39507421 PMC11537994

[220] Jiang Y Liao H-L Chen L-Y. A pan-cancer analysis of SLC12A5 reveals its correlations with tumor immunity. Dis Markers. (2021) 2021:3062606. doi: 10.1155/2021/3062606 34630736 PMC8495467

[221] ESMO-GI. Phase Ib/IIa study to evaluate safety and efficacy of priming treatment with the hedgehog inhibitor NLM-001 prior to gemcitabine and nab-paclitaxel plus zalifrelimab as first-line treatment in patients with advanced pancreatic cancer: NUMANTIA study [WWW Document], n.d. (2024). Available at: https://clin.larvol.com/abstract-detail/ESMO-GI%202024/70897010 (Accessed March 17, 2025).

[222] Goydel RS Rader C. Antibody-based cancer therapy. Oncogene. (2021) 40:3655–64. doi: 10.1038/s41388-021-01782-7 PMC8357052 33947958

[223] Li Z Wang M Yao X Luo W Qu Y Yu D. Development of a novel EGFR-targeting antibody-drug conjugate for pancreatic cancer therapy. Targeted Oncol. (2019) 14:93–105. doi: 10.1007/s11523-019-00615-2 30635821

[224] Forster T Huettner FJ Springfeld C Loehr M Kalkum E Hackbusch M. Cetuximab in pancreatic cancer therapy: A systematic review and meta-analysis. Oncology. (2019) 98:53–60. doi: 10.1159/000501991 31578019

[225] Christenson E Lim SJ Wang H Ferguson A Parkinson R Cetasaan Y. Nivolumab and a CCR2/CCR5 dual antagonist (BMS-813160) with or without GVAX for locally advanced pancreatic ductal adenocarcinomas: Results of phase I study. J Clin Oncol. (2023). doi: 10.1200/JCO.2023.41.4_suppl.730. Advance online publication.

[226] Chen S Li J Dong A Liu Z Zhu M Jin M. Nab-paclitaxel and gemcitabine plus camrelizumab and radiotherapy versus nab-paclitaxel and gemcitabine alone for locally advanced pancreatic adenocarcinoma: a prospective cohort study. J Hematol Oncol. (2023) 16(1):26. doi: 10.1186/s13045-023-01422-8 36941671 PMC10026489

[227] Banchereau J Steinman RM. Dendritic cells and the control of immunity. Nature. (1998) 392:245–52. doi: 10.1038/32588 9521319

[228] University of Pennsylvania. Pilot study of mature dendritic cell vaccination against mutated KRAS in patients with resectable pancreatic cancer (Clinical Trial Registration NCT03592888) [Clinical trial registration]. ClinicalTrials.gov (2024). Available at: https://clinicaltrials.gov/study/NCT03592888.

[229] Hosein AN Dougan SK Aguirre AJ Maitra A. Translational advances in pancreatic ductal adenocarcinoma therapy. Nat Cancer. (2022) 3:272–86. doi: 10.1038/s43018-022-00349-2 35352061

[230] Chen J Sobecki M Krzywinska E Thierry K Masmoudi M Nagarajan S. Fibrolytic vaccination against ADAM12 reduces desmoplasia in preclinical pancreatic adenocarcinomas. EMBO Mol Med. (2024) 16:3033–56. doi: 10.1038/s44321-024-00157-4 PMC11628623 39478152

[231] Tan S Li D Zhu X. Cancer immunotherapy: Pros, cons and beyond. Biomed Pharmacother. (2020) 124:109821. doi: 10.1016/j.biopha.2020.109821 31962285

[232] Akce M Zaidi MY Waller EK El-Rayes BF Lesinski GB. The potential of CAR T cell therapy in pancreatic cancer. Front Immunol. (2018) 9:2166. doi: 10.3389/fimmu.2018.02166 30319627 PMC6167429

[233] Beatty GL Haas AR Maus MV Torigian DA Soulen MC Plesa G. Mesothelin-specific chimeric antigen receptor mRNA-engineered T cells induce anti-tumor activity in solid Malignancies. Cancer Immunol Res. (2013) 2:112. doi: 10.1158/2326-6066.CIR-13-0170 PMC3932715 24579088

[234] Ramalingam PS Premkumar T Sundararajan V Hussain MS Arumugam S. Design and development of dual targeting CAR protein for the development of CAR T-cell therapy against KRAS mutated pancreatic ductal adenocarcinoma using computational approaches. Discover Oncol. (2024) 15:592. doi: 10.1007/s12672-024-01455-6 PMC11511808 39453574

[235] Caruana I Savoldo B Hoyos V Weber G Liu H Kim ES. Heparanase promotes tumor infiltration and antitumor activity of CAR-redirected T lymphocytes. Nat Med. (2015) 21:524–9. doi: 10.1038/nm.3833 PMC4425589 25849134

[236] Clinical Trial: NCT03592888 - My Cancer Genome [WWW Document], n.d. Available at: https://www.mycancergenome.org/content/clinical_trials/NCT03592888/ (Accessed March 16, 2025).

[237] Seo YD Jiang X Sullivan KM Jalikis FG Smythe KS Abbasi A. Mobilization of CD8^+^ T cells via CXCR4 blockade facilitates PD-1 checkpoint therapy in human pancreatic cancer. Clin Cancer Res. (2019) 25:3934–45. doi: 10.1158/1078-0432.CCR-19-0081 PMC6606359 30940657

[238] O’Hara MH O’Reilly EM Varadhachary G Wolff RA Wainberg ZA Ko AH. CD40 agonistic monoclonal antibody APX005M (sotigalimab) and chemotherapy, with or without nivolumab, for the treatment of metastatic pancreatic adenocarcinoma: An open-label, multicentre, phase 1b study. Lancet Oncol. (2021) 22:118–31. doi: 10.1016/S1470-2045(20)30532-5 33387490

[239] Kast V Nadernezhad A Pette D Gabrielyan A Fusenig M Honselmann KC. A tumor microenvironment model of pancreatic cancer to elucidate responses toward immunotherapy. Advanced Healthc Mater. (2023) 12:2201907. doi: 10.1002/adhm.202201907 PMC11468239 36417691

